# ASCL2 Affects the Efficacy of Immunotherapy in Colon Adenocarcinoma Based on Single-Cell RNA Sequencing Analysis

**DOI:** 10.3389/fimmu.2022.829640

**Published:** 2022-06-03

**Authors:** Lei Wu, Shengnan Sun, Fei Qu, Xiuxiu Liu, Meili Sun, Ying Pan, Yan Zheng, Guohai Su

**Affiliations:** ^1^ Research Center of Translational Medicine, Central Hospital Affiliated to Shandong First Medical University, Jinan, China; ^2^ Research Center of Translational Medicine, Jinan Central Hospital, Shandong University, Jinan, China; ^3^ Department of Oncology, Central Hospital Affiliated to Shandong First Medical University, Zhuhai, China; ^4^ Department of Pathology, Central Hospital Affiliated to Shandong First Medical University, Jinan, China; ^5^ Department of Oncology, Zhuhai People’s Hospital (Zhuhai Hospital Affiliated With Jinan University), Zhuhai, China

**Keywords:** colon adenocarcinoma, single-cell RNA-seq, microsatellite stable, microsatellite instability-high, immunotherapy

## Abstract

Colon adenocarcinoma (COAD) is one of the leading causes of cancer-associated deaths worldwide. Patients with microsatellite instability-high (MSI-H) tumors were shown to highly benefit from immune checkpoint inhibitors (ICIs) than patients with microsatellite stable (MSS) tumors. Furthermore, the infiltration of immune cells and the expression of cancer stem cells (CSCs) in COAD were associated with the anti-tumor immune response. However, the potential mechanisms showing the relationship between microsatellite instability and CSCs or tumor-infiltrating immune cells (TIICs) have not been elucidated. Accumulating evidence reveals that achaete-scute family bHLH transcription factor 2 (ASCL2) plays a crucial role in the initiation and progression of COAD and drug resistance. However, the specific biological functions of ASCL2 in COAD remain unknown. In this study, we performed weighted gene co-expression network analysis (WGCNA) between MSS and MSI-H subsets of COAD. The results revealed that ASCL2 was a potential key candidate in COAD. Subsequently, the single-cell RNA-seq revealed that ASCL2 was positively associated with CSCs. Further, ASCL2 was shown to indirectly affect tumor immune cell infiltration by negatively regulating the expression of DUSP4. Finally, we inferred that the immunotherapy-sensitive role of ASCL2/DUSP4 axis on COAD is partly attributed to the activation of WNT/β-catenin pathway. In conclusion, this study revealed that ASCL2 was positively correlated to CSCs and tumor immune infiltration in COAD. Therefore, ASCL2 is a promising predictor of clinical responsiveness to anti-PD-1/PD-L1 therapy in COAD.

## Introduction

Colon adenocarcinoma (COAD) is one of the leading causes of cancer-associated deaths worldwide (1). Conventional therapy for COAD includes surgery, targeted therapy, and chemotherapy. Despite progress in the treatment of COAD, the prognosis remains poor (2). Therefore, novel therapeutic strategies with high efficacy are urgently required.

An increasing number of new effective anti-tumor therapies have been developed (3). The activation of anti-tumor immune surveillance by immune checkpoint inhibitors is an attractive strategy (4). Immune checkpoint inhibitors (ICIs) such as programmed cell death-1 (PD-1) and programmed death-ligand 1 (PD-L1) inhibitors have made a significant breakthrough in COAD treatment (5).

Microsatellites are highly polymorphic DNA sequences located throughout the human genome. They display a high degree of inter-individual variation (6). Microsatellite instability (MSI) may occur due to mutations or epigenetic changes in DNA mismatch repair (MMR) genes, thus impairing the function of the DNA MMR system (7). High microsatellite instability (MSI-H) is the hallmark of tumors with a mismatch DNA repair deficiency (dMMR) (8). Immune checkpoint inhibitors were shown to be effective in dMMR/MSI-H subsets of COAD. However, ICIs were ineffective in microsatellite instability-low or microsatellite stable (MSI-L/MSS) and mismatch repair proficient (pMMR) subsets (9). However, the specific mechanism by which MSI-H/dMMR influences the efficacy of ICIs in COAD remains unknown.

Achaete-scute family bHLH transcription factor 2 (ASCL2) is a transcription factor that is over-expressed in colon cancer. Previous studies have shown that ASCL2 can affect anti-tumor drug sensitivity (10). Furthermore, recent studies have revealed that ASCL2 is associated with immune infiltration in colorectal cancer. Microsatellite stable colorectal cancer samples overexpressing ASCL2 were shown to have low CD8^+^ T cell infiltration (11). Moreover, a vaccine targeting ASCL2 was shown to affect the efficacy of anti-PD-1 in colorectal cancer (12). Nevertheless, the potential mechanism behind the association of ASCL2 with immune infiltration and immunotherapy in COAD remains unknown.

In this study, we performed multiple methods between dMMR/MSI-H and pMMR/MSS of COAD. Meanwhile, ASCL2 might have an effect on tumor immune cell infiltration through an indirect mechanism by negatively regulating the expression of DUSP4. Finally, we inferred that immunotherapy-sensitive role of ASCL2/DUSP4 axis on COAD is attributed, at least partly, to the activation of WNT/β-catenin pathway. In brief, our findings elucidated that ASCL2 correlated with CSCs and tumor immune infiltration in COAD. ASCL2 might serve as a promising predictor of clinical responsiveness to anti-PD-1/PD-L1 therapy in COAD.

## Materials and Methods

### Cell Culture

Human colon cancer cell line, HCT116 cells, were cultured in RPMI-1640 medium supplemented with 10% fetal bovine serum (FBS). The cells were grown in a humidified atmosphere supplemented with 5% CO_2_ at 37°C.

### Plasmid and siRNA Transfection

Two different small interfering RNAs (siRNAs) for specific inhibition of ASCL2 expression and a negative control siRNA were synthesized by Research cloud biology Co., Ltd. (Shandong, China). The ASCL2 gene was inserted into pcDNA3.1 by Boshang Biotechnology (Shandong, China). The empty vector was used as the negative control. Subsequently, exponentially growing untreated cells were plated 24 h before transfection. ASCL2-specific siRNA and pcDNA3.1-ASCL2 were transiently transfected with HCT116 cells using lipofectamine 2000 (Invitrogen, USA), and the cells were subsequently cultured for 48 hours. After that, total proteins were extracted for western blot analysis.

### Western Blotting

Total protein was extracted from the HCT116 cells using RIPA buffer (Beyotime, China). Total proteins were separated in SDS-PAGE and then transferred onto PVDF membranes. After blocking with 5% nonfat milk for two hours at room temperature, the membranes were incubated overnight with the primary antibodies ASCL2 (1:1000, Abways Technology), DUSP4 (1:1000, ABclonal) and c-myc(1:1000, Proteintech) at 4°C. Subsequently, the membranes were incubated with the corresponding secondary antibodies, and visualization was done using enhanced chemiluminescence (ECL, Thermo Fisher Scientific).

### Immunohistochemistry Analysis

For IHC analysis, the COAD and adjacent tissue paraffin-embedded slides were deparaffinized and rehydrated using xylene and a graded series of ethanol (100%, 95%, 80%, 75%), then washed with PBS three times for 5 minutes each time. Then, EDTA antigen restore solution was used to repair antigens on slices in a microwave oven at the condition of high heat for 5 min, heat preservation for 10 min, and high heat for 5 min followed by natural cooling, and washed with PBS three times for 5 minutes each time. Then immersed in 3% H_2_O_2_ solution at room temperature to abrogate endogenous peroxidase activity. The slides were incubated in 5% BSA to block non-specific binding of antibody for 1 hour, then incubated in a humidified chamber overnight at 4°C with the primary antibodies anti-ASCL2 (1:200 dilution; Bioss, Beijing, China). After PBS washes, the slides were subsequently incubated with goat anti-rabbit HRP secondary antibody for 60 min at room temperature, followed by PBS washes again. For a color reaction, slides were incubated with the DAB solution (Biyuntian Biotechnology Co., Ltd.). Subsequently, the slides were then counterstained with hematoxylin, dehydrated with graded alcohol series, covered-slipped with neutral balsam.

### Data Acquisition and Selection

The transcriptome profiles of COAD were acquired from the TCGA database (https://portal.gdc.cancer.gov/), containing 473 COAD tissues and 41 non-tumor tissues. Microarray dataset GSE39582 was downloaded from the gene expression omnibus (GEO) database (https://www.ncbi.nlm.nih.gov/geo/). The GPL570 dataset contained 519 samples, including 444 COAD tissues with pMMR and 75 COAD tissues with dMMR. The single-cell RNA sequencing data of human colon cancer samples (accession number GSE166555) were generated using 10× genomics. For accurate results, we used MSI patient as a benchmark, selected data source of C18.9 without lymph node metastasis samples for subsequent analysis.

### Construction of Weighted Gene Co-Expression Network Analysis

The raw microarray data, GSE39582, were processed and normalized using R software (version 4.0.5) to identify significant mRNAs associated with the MMR status of COAD. The genes were ranked by SD values from large to small, and the top 25% genes were chosen for WGCNA analysis using R package “WGCNA” (13). β = 3 was selected as the soft threshold. After that, the minimum number of genes in a module was set at 100, with a total of six modules. Then, the Pearson correlation coefficient was calculated to determine the degree of association between the MMR status and the module. In general, the module with the absolute correlation coefficient ranked first among all the selected modules was considered related to the clinical trait. Finally, 736 genes that were most relevant to MSS/pMMR and MSI-H/dMMR were identified in the blue module.

### Identification of Differentially Expressed Genes Between MSS/pMMR COAD and MSI-H/dMMR COAD

Differentially expressed genes (DEGs) were identified by comparing MSI-H/dMMR COAD tissues with MSS/pMMR COAD tissues using the R software. Adjusted P value < 0.05 and |log FC| > 1 were set as the cut-off values. The analysis revealed 179 DEGs, including 91 upregulated and 88 downregulated DEGs.

### Functional and Pathway Enrichment Analysis

Kyoto Encyclopedia of Genes and Genomes (KEGG) analysis was performed to annotate pathways in the KEGG pathway database and identify the potential biological mechanism of hub genes,. KEGG pathway enrichment was performed using R package “clusterProfiler” with *P* < 0.05 (R version 4.0.5) (14).

### Identification of Hub Genes From Protein–Protein Interaction Network

The STRING database is a functional protein association database for forecasting protein-protein interactions (15). Cytoscape (version 3.8.2) was used to display the PPI network after the DEGs were uploaded to STRING (16). CytoHubba (version 0.1) was used to detect candidate hub genes based on the density of the maximum neighborhood component (DMNC) algorithm (17).

### Processing of Single-Cell RNA-Seq Data

Single-cell RNA (scRNA) sequencing data was extracted from the human derived COAD with MSI and MSS group. For accurate results, we used MSI patient as a benchmark, selected data source of C18.9 without lymph node metastasis samples for subsequent analysis. The Seurat package in R software was used to organize and analyze the scRNA-seq data (18). Quality control was carried out as follows: 1) Genes expressed in less than ten cells were excluded; 2) cells expressing less than 200 unique gene counts were excluded. After that, the top 1000 highly variable genes were selected using “vst” of Seurat. Dimensionality reduction of scRNA-seq data was performed using the t-distributed stochastic neighbor embedding (tSNE) algorithm (19). An adjusted P value < 0.05, | log FC | > 0.5, pct 1≥0.5 and pct 2 < 0.5 were considered the cut-off values for identifying marker genes. In addition, different cell clusters were annotated using the SingleR algorithm (20).. Finally, the CellMarker database was used for manual verification and correction (21).

### Correlation Analysis of ASCL2/DUSP4 With MSI and Immune Infiltration Cells in Pan-Cancer

The correlation between ASCL2/DUSP4 and MSI score and immune cells in several cancer types were analyzed in “Gene+” module of Sangerbox. In terms of colon cancer, to make reliable immune infiltration estimations, we utilized the immunedeconv, an R package which integrates TIMER algorithms. It mainly calculated the infiltration scores of six immune infiltration cells: CD4+ T cell, CD8+T cell, neutrophil, dendritic cell, B cell and macrophage.

### Data Acquisition of Spatial Transcriptomic Dataset

The spatial transcriptomics dataset for colon cancer was obtained from the 10X Genomics website. The Visium Gene Expression Library (T1T2-E8) was prepared as described in the Visium Spatial Reagent Kits User Guide (CG000239 Rev D). Sequencing data were processed using Space Ranger.

### Gene Set Enrichment Analysis

The GSEA was performed using the GSEA v4.1.0 software. COAD samples were classified into high-and low-expression groups using the median expression values of ASCL2 and DUSP4 as the cut-off values. The procedure followed the detailed protocol from the Broad Institute Gene Set Enrichment Analysis website (22). A gene set was considered significantly enriched at NOM p-value less than 0.05 and FDR q-value less than or equal to 0.1 (23).

### Wnt/β-Catenin Pathway Inhibitor Response Predictions

To predict drug-sensitivity of ASCL2 and DUSP4, the pRRophetic package of R software was implemented to extrapolate half-maximal inhibitory concentration (IC50) values by building ridge regression model with ten-fold cross-validation. Wnt/β-catenin pathway inhibitor drug XAV939 and its genetic profiles were obtained from the Genomics of Drug Sensitivity in Cancer (GDSC).

### Statistical Analyses

The boxplots for the two groups were analyzed using Wilcoxon test. Further, correlation analysis was carried out using Spearman correlation test. For RNAseq data of Pan-cancer, expression levels were TPM-normalized. The statistical analyses were performed using R software (version 4.0.5). A p-value <0.05 was considered statistically significant.

## Results

### Weighted Co-Expression Network Construction

**Figure 1:**
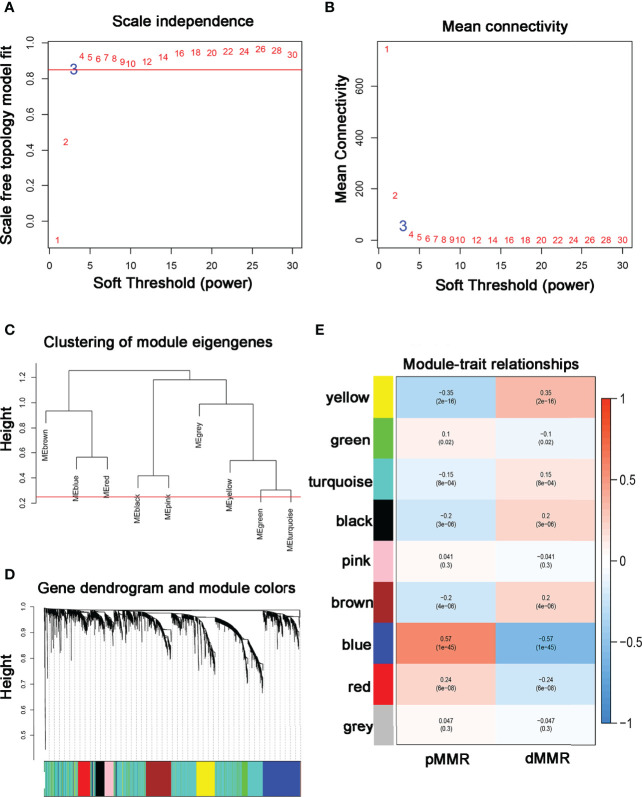
Construction of co-expression modules using WGCNA **(A, B)** The most fit soft-thresholding power of WGCNA was 3. **(C)** Hierarchical clustering tree showing co-expression modules. **(D)** The cluster dendrogram of genes in GSE39582. **(E)** Heatmap showing the correlation between 9 modules and MMR status of COAD. The blue module showed the strongest correlation with pMMR and dMMR status (p = 1×10^-45^, r = -0.57).

Data obtained from the GSE39582 were preprocessed using R to obtain 21,654 genes used to construct the WGCNA networks. A total of 5,414 genes were used for cluster analysis using the WGCNA package. β = 3 was set as the soft threshold power (**Figures 1A, B**). The cluster dendrogram constructed based on the selected threshold identified nine color modules (**Figures 1C, D**). There were 195 genes in the black module, 736 genes in the blue module, 527 genes in the brown module, 278 genes in the green module, seven genes in the grey module, 187 genes in the pink module, 229 genes in the red module, 2196 genes in the turquoise module, and 419 genes in the yellow module. Finally, the mRNAs in the nine color modules were used to analyze the relationship between the modules and the traits (pMMR status and dMMR status). The blue module showed a high correlation with the pMMR and dMMR status compared with other modules (**Figure 1E**), indicating that genes in the blue module play significant roles in the treatment of COAD patients with PD-1/PD-L1 inhibitors. Details of specific gene names in blue module can be found in **Supplementary Table 1**.

### Identification of Hub Genes and Functional Enrichment Analysis

**Figure 2:**
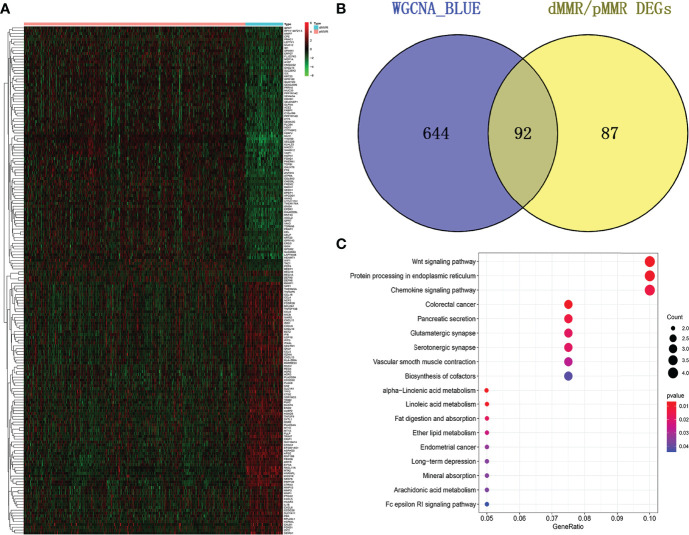
Overlapping genes and enrichment analysis**. (A)** Heatmap of DEGs in the GSE39582 dataset. **(B)** A Venn diagram showing overlapping genes between dMMR/pMMR DEGs and WGCNA-MEblue genes. **(C)** The 18 significantly enriched pathways associated with the overlapping genes as determined through KEGG enrichment analysis.

The microarray dataset, GSE39582, revealed 179 DEGs (**Figure 2A**; **Supplementary Table 2**). After that, the 736 genes identified in the blue module were intersected with the 179 DEGs. In total, 92 common genes were identified through a comprehensive analysis of the two datasets (**Figure 2B**). Further, the KEGG pathway analysis revealed 18 signaling pathways that were concentrated on the overlapped 92 DEGs. The genes were mainly enriched in the Wnt signaling pathway, protein processing in the endoplasmic reticulum, chemokine signaling pathway, and colorectal cancer-related pathways (**Figure 2C**).

### PPI Network Construction, Hub Gene Selection, and Hub Gene Expression in Pan-Cancer

**Figure 3:**
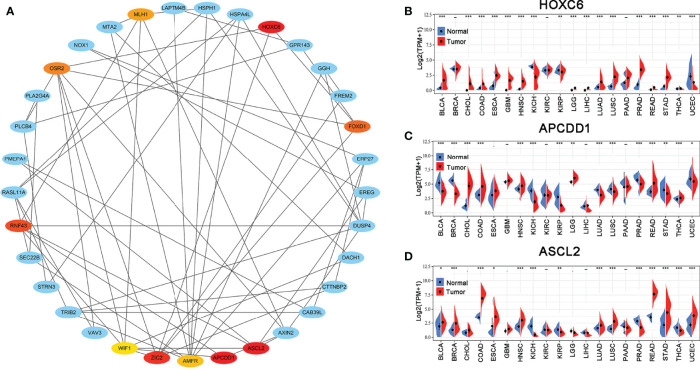
PPI network and hub gene selection. **(A)** Top ten hub genes identified using the cytoHubba from Cytoscape based on DMNC scores. The darker the color, the higher the score. The expression levels of **(B)** HOXC6, **(C)** APCDD1, and **(D)** ASCL2 in multiple human cancers based on TCGA cancer and normal data. *p < 0.05, **p < 0.01, and ***p < 0.001.

The PPI network of the hub genes, including 33 nodes and 64 edges was constructed. Subsequently, the top ten hub genes (HOXC6, APCDD1, ASCL2, ZIC2, RNF43, FOXD1, OSR2, MLH1, AMFR, and WIF1) were selected based on the DMNC scores *via* the cytoHubba (**Figure 3A**). The scoring file is detailed in **Supplementary Table 3**. Immediately, we analyzed the expression of the top three genes, which tie for first place (HOXC6, APCDD1, and ASCL2) in multiple cancer types to explore possible roles of the genes in carcinogenesis. The results revealed that HOXC6, APCDD1, and ASCL2 were significantly upregulated in COAD compared with the adjacent non-tumor tissues, as shown in **Figures 3B–D**. Moreover, ASCL2 was highly expressed in COAD compared with HOXC6 and APCDD1 in multiple tumor types. As shown in **Supplementary Figures 4A, B**, high mRNA expression of ASCL2 was detected in human colon cancer cell lines and colon cancer tissues. Meanwhile, by immunohistochemistry, colon cancer tissues were found to express stronger ASCL2 than normal colon tissues (**Supplementary Figures 4C, D**). Taken together, ASCL2 may be a crucial regulator of carcinogenesis in COAD.

### Single-Cell RNA-Seq Profiling of the Microsatellite Status

**Figure 4:**
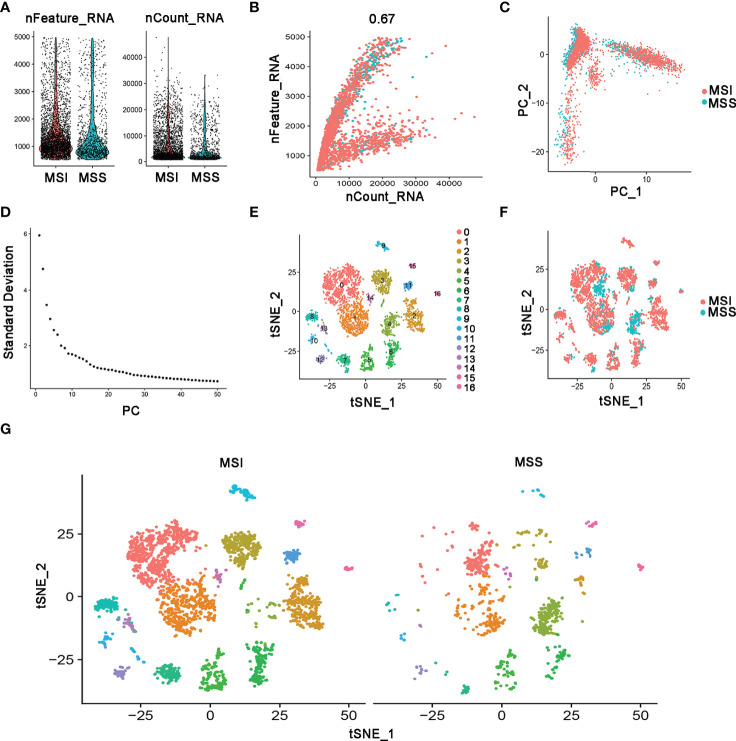
Identification of 17 cell clusters in COAD by single-cell sequencing analysis. **(A)** A total of 4,196 cells identified after quality control. **(B)** Genes correlated with sequencing depth, with a Pearson’s correlation coefficient of 0.67. **(C)** Comparison of batch effects between groups. **(D)** The 50 PCs with P value < 0.05 identified using PCA. **(E)** The 17 cell clusters classified using the tSNE algorithm. **(F, G)** The distribution of cells between MSS and MSI in COAD patients.

The single-cell RNA sequencing data of human colon cancer samples were generated using 10× genomics. For accurate results, we used MSI patient as a benchmark, selected data source of C18.9 without lymph node metastasis samples for subsequent analysis. The pathological stage and grade of the MSI patient were pT1 N0 and G2, respectively. The pathological stage and grade of the MSS patient were pT3 N0 and G2. Microsatellite status was determined by immunohistology for mismatch repair protein deficiency. We analyzed the single-cell RNA-seq data to identify the molecular features in the microsatellite status of colon cancer. A total of 4196 cell samples were acquired from two groups consisting of MSS-COAD and MSI-COAD. The number of detected genes and the sequencing count of each cell were illustrated in a quality control chart, as shown in **Figure 4A**. The number of genes detected was positive correlated with the sequencing depth using Pearson’s R = 0.67 (**Figure 4B**). In addition, the cells were mapped into two dimensions based on PC_1 and PC_2 components. The two correct independent cell subpopulations indicated the preferable clustering efficiency during the principal component analysis (PCA) procedure (**Figure 4C**). A total of 50 principal components (PCs) were selected for subsequent analysis (**Figure 4D**). Afterward, the tSNE algorithm was applied, and cells in COAD with MSS or MSI were classified into 17 separate clusters (**Figure 4E**). There were significant differences between the two groups in terms of the distribution of cells (**Figures 4F, G**).

### Single-Cell RNA-Seq Screening of Microsatellite Status-Associated Marker Genes

**Figure 5:**
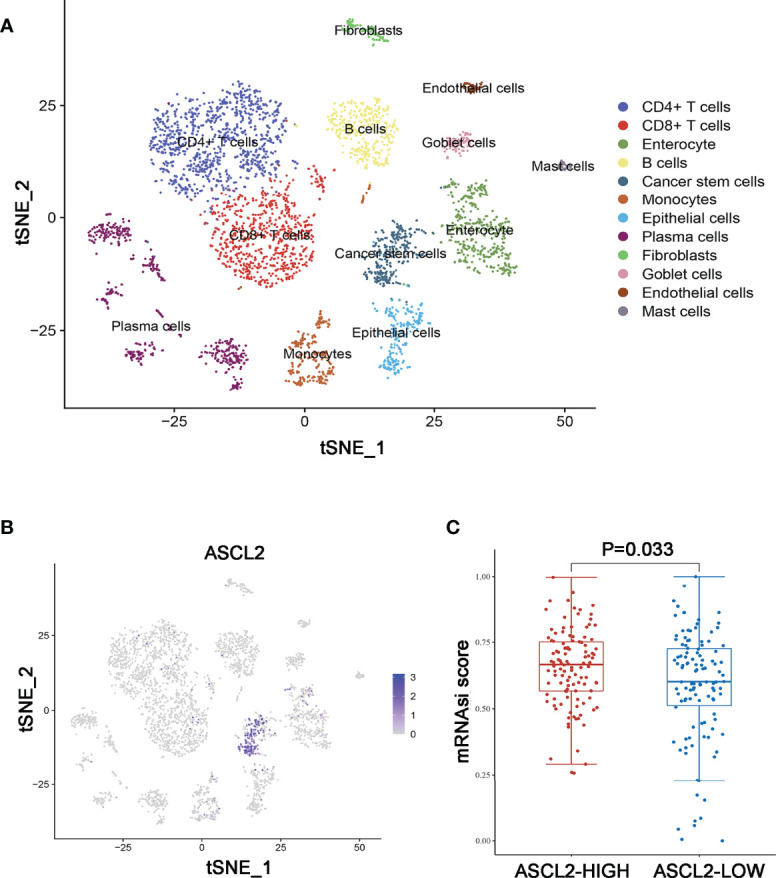
Annotation of MSS/MSI COAD single-cell data set. **(A)** The 17 cell clusters were annotated into major types using singleR and CellMarker. **(B)** t-SNE plots of single-cell sequencing results showing that ASCL2 was expressed in cancer stem cells. **(C)** The distribution of mRNAsi in different groups, where the abscissa represents samples from different groups, and the ordinate represents the distribution of mRNAsi scores. The red dots represent the group with high expression of ASCL2, while blue dots represent the group with low expression of ASCL2.

Further, a total of 3911 unique marker genes from all 17 clusters were identified. Then, the adjusted P-value < 0.05, | log FC] | > 0.5, pct 1≥0.5 and pct 2 < 0.5 were considered the cut-off criteria for further screening to identify relevant marker genes. Ultimately, a total of 3166 marker genes (1968 unique genes) were identified for further analysis. The clusters were annotated using singleR and CellMarker according to the expression patterns of the marker genes. Cluster 0, containing 951 cells, was annotated as CD4^+^ T cells, clusters 1 and 14, containing 728 cells, were annotated as CD8^+^ T cells, cluster 2, containing 436 cells, was annotated as enterocytes, cluster 3, containing 385 cells, was annotated as B cells, cluster 4, containing 287 cells, was annotated as cancer stem cells, cluster 5, containing 284 cells, was annotated as monocytes, cluster 6, containing 246 cells, was annotated as epithelial cells, clusters 7, 8, 10, 12 and 13, containing 575 cells, were annotated as plasma cells, cluster 9, containing 111 cells, was annotated as fibroblasts, cluster 11, containing 98 cells, was annotated as goblet cells, cluster 15, containing 51 cells, was annotated as endothelial cells, while cluster 16, containing 44 cells, was annotated as mast cells (**Figure 5A**). The cells, especially the CD4^+^ T cells, CD8^+^ T cells, B cells, cancer stem cells, and enterocytes, were significantly altered in the MSI group compared to the MSS group. Then, we examined the expression of ASCL2 in the above 17 different cell types. As illustrated in **Figure 5B**, ASCL2 was highly expressed in cancer stem cells (cluster 4). mRNA expression-based stemness index (mRNAsi) is involved in maintaining cancer stem-like properties in specific tumor types. The OCLR algorithm constructed by Malta et al. (24) revealed that mRNAsi in the ASCL2-high expression group was significantly higher than that of the ASCL2-low expression group (**Figure 5C**). In previously **Figures 4F, G**, we identified that the cancer stem cells in the MSS group were significantly increased compared with the MSI group. This implies that ASCL2-related cancer stem cell signature could affect the efficacy of colon cancer immunotherapy.

### Identification of DEGs in COAD

The genes were annotated based on Ensembl and the TCGA databases. Based on the given threshold (|log FC| >2 and adjust P -value < 0.05), 1570 differentially expressed genes (DEGs) were identified between 473 COAD tissues and 41 normal tissues, of which 582 DEGs were upregulated and 988 DEGs were downregulated (**Supplementary Figure 1**; **Supplementary Table 4**).

### The Enrichment of Candidate Genes in T Cells

**Figure 6:**
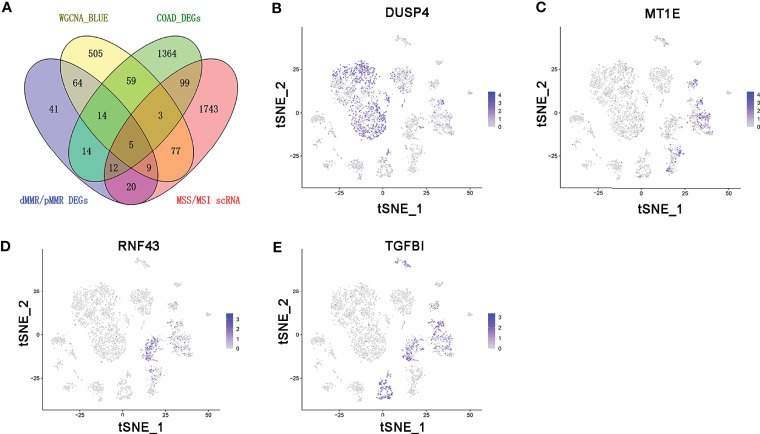
The expression profile of DUSP4, MT1E, RNF43, and TGFBI is displayed on tSNE projection. **(A)** The Venn diagram showing the top 5 common genes (ASCL2, DUSP4, MT1E, RNF43 and TGFBI) included in 4 different gene expression datasets. **(B)** t-SNE plots of single-cell sequencing results showing that DUSP4 was expressed in CD4+ T cells (cluster 0) and CD8+ T cells (cluster 1 and 14). **(C)** t-SNE plots of single-cell sequencing results demonstrating that MT1E was expressed in enterocyte (cluster 2), epithelial cells (cluster 6), fibroblasts (cluster 9) and goblet cells (cluster 11). **(D)** t-SNE plots of single-cell sequencing results indicating that RNF43 was expressed in enterocyte (cluster 2) and cancer stem cells (cluster 4). **(E)** t-SNE plots of single-cell sequencing results demonstrating that TGFBI was expressed in enterocyte (cluster 2), cancer stem cells (cluster 4), monocytes (cluster 5), fibroblasts (cluster 9) and goblet cells (cluster 11).

The persistence of T cells *in vivo* may play a paramount role in the efficacy of immunotherapy with anti-PD-1/PD-L1. The Venn diagram identified five common genes (ASCL2, DUSP4, MT1E, RNF43, and TGFBI) in the four different gene expression datasets obtained by diverse data analysis methods, such as WGCNA, differential gene expression analysis, and single-cell sequencing analysis (**Figure 6A**). As shown in **Figures 6B–E**, only DUSP4 was highly expressed in CD4+ T cells (cluster 0) and CD8+ T cells (clusters 1 and 14). Based on this finding, we hypothesized that DUSP4 could enhance T cell expression, thus improving immunotherapy with anti-PD-1/PD-L1.

### Correlation Between the Expression of ASCL2 and DUSP4 With MSI and Immune Landscape in Pan-Cancer

**Figure 7:**
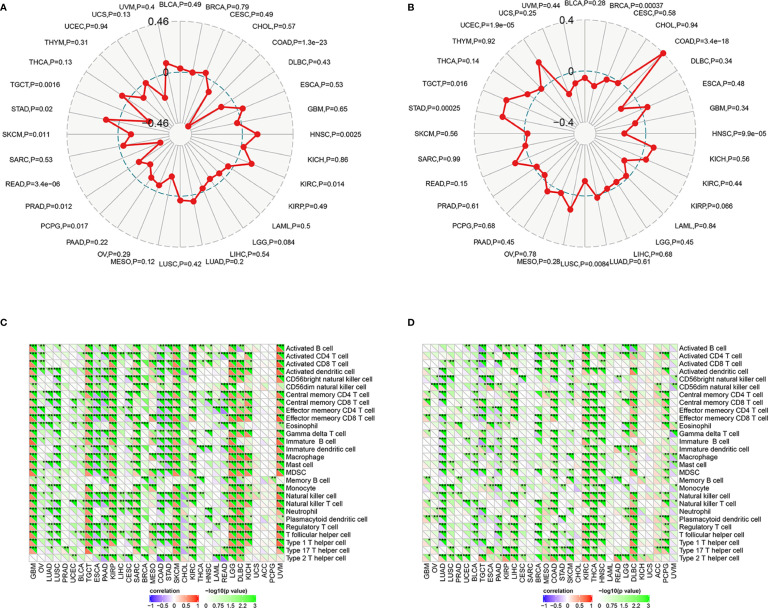
Association of ASCL2 and DUSP4 expression with MSI and immune infiltration in Pan-cancer. Radar plots showing the association of **(A)** ASCL2 and **(B)** DUSP4 expression with MSI across 32 different cancer types. The red curve indicates the correlation coefficient. **(C)** Association of immune infiltration with ASCL2. **(D)** Association of immune infiltration with DUSP4. Positive correlations are shown in red, whereas negative correlations are shown bluish violet. *p < 0.05, **p < 0.01, and ***p < 0.001.

Microsatellite instability occurs in multiple cancer types and acts as a predictive biomarker for immunotherapy efficacy. Therefore, we determined the correlation between the expression of ASCL2 and DUSP4 with MSI in 32 cancer types. As shown in **Figure 7A**, the expression of ASCL2 was negatively correlated with MSI in COAD. In contrast, the expression of DUSP4 was positively correlated to MSI in COAD (**Figure 7B**). Notably, ASCL2 and DUSP4 had the highest association with MSI in COAD compared to the other cancer types. On the other hand, tumor-infiltrating immune cells (TIICs) are components of the tumor microenvironment that influence sensitivity to immunotherapy in multiple cancers. Hence, we explored the correlation between the expression of ASCL2 and DUSP4 with TIICs in Pan-cancer. The results revealed that ASCL2 was highly correlated to the infiltration levels of CD4^+^ T cells, CD8^+^ T cells, B cells, neutrophils, and dendritic cells in the vast majority of cancer types. Furthermore, the expression of ASCL2 was inversely correlated to the immune cell infiltration level of CD4^+^ T cells, CD8^+^ T cells, B cells, neutrophils, and dendritic cells in COAD (**Figure 7C**). However, data from DUSP4 with diametrically opposed results in COAD (**Figure 7D**). Hence, the above results suggested that ASCL2 and DUSP4 expression were closely related to the extent of immune infiltration in multiple cancer types, including COAD. Moreover, there might be a mutually antagonistic effect between ASCL2 and DUSP4 in COAD.

### Relationship Between the Immune Cell Infiltrates and the Expression of ASCL2 and DUSP4 in COAD Patients

**Figure 8:**
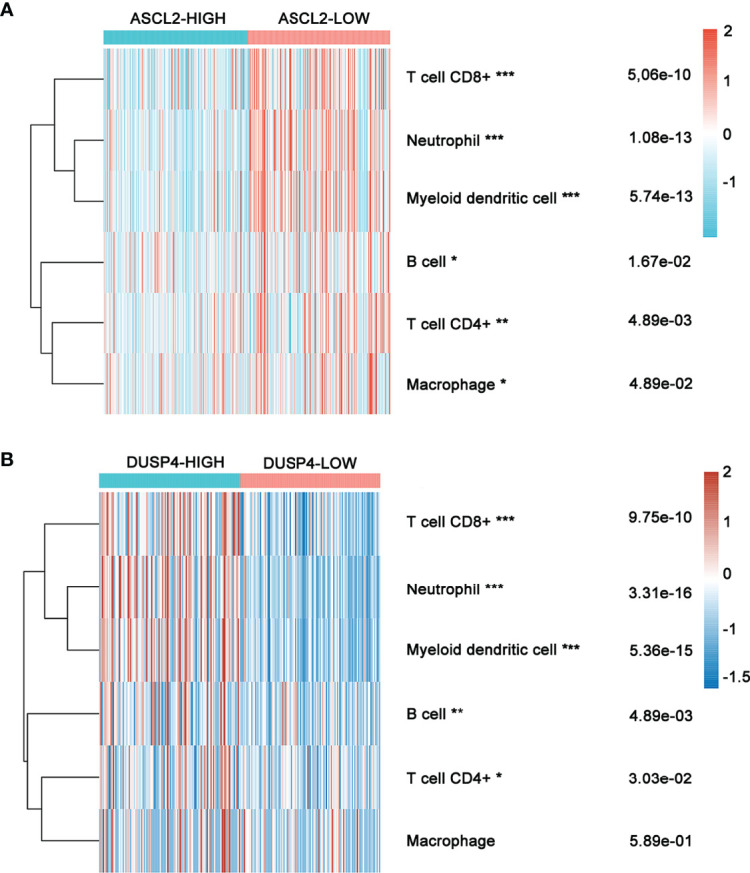
Heatmap showing immune cell infiltration in COAD patients with high or low ASCL2 and DUSP4 expression. **(A)** Comparison of immune infiltration between the high-and low-expression of ASCL2 in COAD patients. **(B)** Comparison of immune infiltration between the high-and low-expression of DUSP4 in COAD patients. *p < 0.05, **p < 0.01, and ***p < 0.001.

The expression of six main infiltrating immune cell types, (CD8^+^ T cells, CD4^+^ T cells, B cells, neutrophils, dendritic cells, and macrophages) was determined and compared between ASCL2 and DUSP4 in COAD patients. The ASCL2 low expression group contained a higher proportion of CD8^+^ T cells (P = 5.06e-10), neutrophils (P = 1.08e-13), dendritic cells (P =5.74e-13), B cells (P=1.67e-02), CD4^+^ T cells (P = 4.89e-03), and macrophages (P = 4.89e-02) compared with the ASCL2 high expression group (**Figure 8A**). Conversely, the DUSP4 high expression group had a higher proportion of CD8^+^ T cells (P = 9.75e-10), neutrophils (P = 3.31e-16), dendritic cells (P =5.36e-15), B cells (P=4.89e-03), and CD4^+^ T cells (P = 3.03e-02) compared with the DUSP4 low expression group. However, there were no significant differences observed in the expression of macrophages (P = 5.89e-01) (**Figure 8B**).

### Transcription Factor ASCL2 Regulated the Expression Level of DUSP4

**Figure 9:**
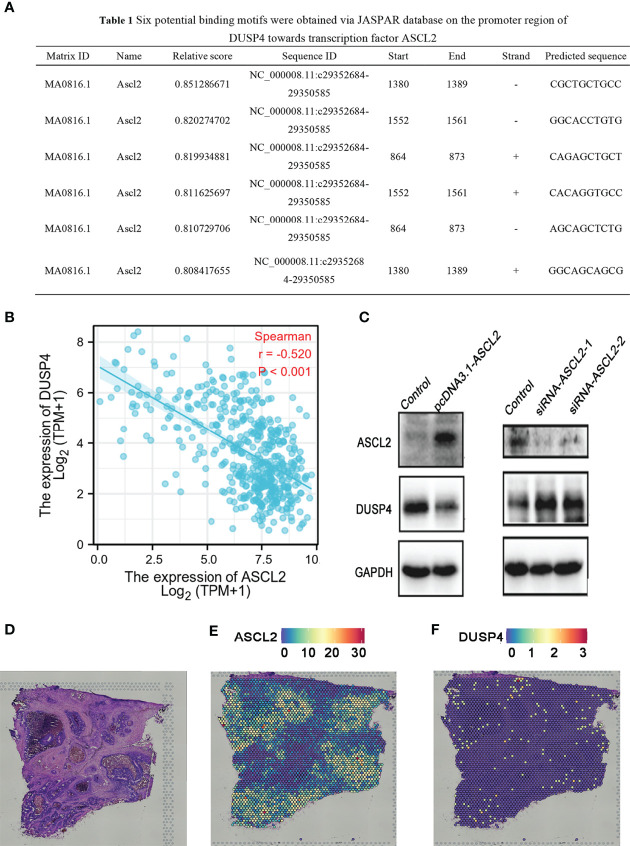
DUSP4 expression was negatively regulated by ASCL2 in COAD samples. **(A)** Predicted ASCL2 binding sites within the region of DUSP4 promoter. **(B)** A scatter plot showing the association of ASCL2 with DUSP4 in COAD. **(C)** ASCL2 was upregulated in pcDNA3.1-ASCL2 group. DUSP4 expression was lower in pcDNA3.1-ASCL2 group than in the control group. ASCL2 expression was lower in si-ASCL2-1 and si-ASCL2-2 groups compared to control group. The decrease in ASCL2 expression was accompanied an increase in DUSP4 expression. **(D)** HE staining of colorectal cancer sections. Spatial transcriptomics expression of **(E)** ASCL2 and **(F)** DUSP4 in cancer tissue. Each dot represents a different locus at which gene expression was profiled.

The scRNA-seq analysis showed no significant enrichment of ASCL2 in CD4^+^ T cells and CD8^+^ T cells. However, the expression of ASCL2 was highly associated with the infiltration levels of CD4+ T cells and CD8+ T cells in various tumors, including COAD. JASPAR is a database for eukaryotic transcription factor binding profiles (25). The JASPAR database predicted six potential binding motifs on the promoter region of DUSP4 for binding the transcription factor ASCL2 (**Figure 9A**). Correlation analysis of ASCL2 and DUSP4 showed a negative correlation between ASCL2 and DUSP4 (r = -0.520, p<0.001) (**Figure 9B**). Further, we knocked down the expression or elevated expression of ASCL2 using siRNA or pcDNA3.1-ASCL2 transfection into HCT116 cells to validate the results. As shown in **Figure 9C**, ASCL2 inhibition by siRNA or overexpression using a pcDNA3.1-ASCL2 plasmid was shown to increase or decrease the expression of DUSP4, respectively. These results suggested that ASCL2 negatively regulates DUSP4. Next, we carried out spatial transcriptomics analysis for the colon cancer tissues (**Figure 9D**). The results showed the enrichment of ASCL2 in colon cancer tissue is higher, while enrichment of DUSP4 is lower (**Figures 9E, F**). The gene expression patterns of ASCL2 and DUSP4 revealed by the spatial transcriptomics were consistent with the gene microarrays.

### GSEA and Drug IC50 Values Analysis of ASCL2 and DUSP4 in COAD

**Figure 10:**
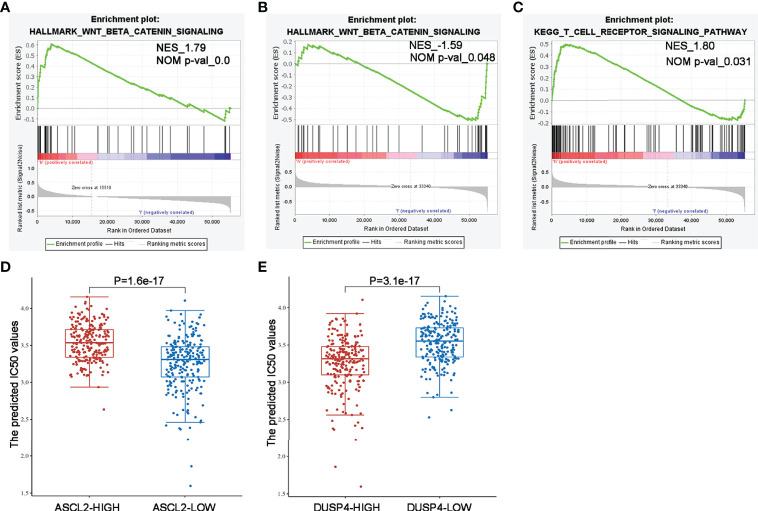
GSEA enrichment and drug IC50 values analysis of ASCL2 and DUSP4 in COAD **(A)** WNT/β-catenin pathway was enriched in ASCL2-related COAD in the Hallmark category. **(B, C)** WNT/β-catenin pathway and T cell receptor signaling were differentially enriched in DUSP4-related COAD in the Hallmark/KEGG category. NES, normalized enrichment score. **(D)** IC_50_ values of Wnt pathway inhibitor was lower in ASCL2 low expression group compared to ASCL2 high expression group. **(E)** IC_50_ values of Wnt pathway inhibitor was higher in DUSP4 low expression group compared to DUSP4 high expression group.

We performed GSEA comparing colon cancer samples with high expression and low expression of ASCL2 and DUSP4 using TCGA dataset to identify pathways correlated with ASCL2 and DUSP4. The GSEA results revealed that ASCL2 and DUSP4 were all enriched in the WNT/β-catenin pathway. Further, as shown in **Figure 10A**, ASCL2 was positively associated with the WNT/β-catenin pathway. However, DUSP4 was negatively associated with the WNT/β-catenin pathway (**Figure 10B**). In addition, DUSP4 was positively correlated with the T cell receptor signaling pathways (**Figure 10C**). XAV939 is an inhibitor of WNT/β-catenin pathway, to investigate the effect of ASCL2 and DUSP4 in WNT/β-catenin pathway, the IC_50_ values of XAV939 on ASCL2 and DUSP4 were calculated. The prediction process was implemented by R package “pRRophetic” where the samples’ half-maximal inhibitory concentration (IC50) was estimated by ridge regression and the prediction accuracy. The results of the IC_50_ values indicated a significant increase in the drug sensitivity of both ASCL2-LOW and DUSP4-HIGH to WNT pathway inhibitor in colon cancer (**Figures 10D, E**). Finally, we transfected ASCL2 over-expression and siRNA plasmids in colon cancer cells, and found ASCL2 positively regulates the expression of c-Myc expression, which is a key gene of the Wnt signaling pathway by western blot (**Supplementary Figure 3**). These results showed that the ASCL2 and DUSP4 were essential effectors in the WNT/β-catenin pathway.

## Discussion

Colon adenocarcinoma patients, especially those with dMMR/MSI-H, show significantly higher sensitivity to ICIs than COAD patients with pMMR/MSS (26, 27). However, metastatic colon cancer patients with MSI-H/dMMR treated with anti-PD-1 show an overall response rate (ORR) of about 33% (28). Therefore, it is important to investigate the underlying molecular mechanisms behind immunotherapy sensitivity. In this study, the differential gene expression and WGCNA analysis conducted between dMMR/MSS and pMMR/MSI-H of COAD revealed 92 hub genes. Furthemore, the PPI network and contrast analysis of the hub genes revealed that ASCL2 was highly correlated with the microsatellite status and abnormal expression of the hub genes. Therefore, we hypothesized that ASCL2 might serve an important role in regulating microsatellite instability status and immunotherapy sensitivity in COAD.

The single-cell RNA-seq analysis revealed that the number of CD4+ T cells, CD8+ T cells, B cells, and cancer stem cells (CSCs) were altered significantly in the MSI group compared with the MSS group. Moreover, ASCL2 was highly expressed in CSCs. Accumulating evidence reveals that CSCs in tumors contributes to chemo and radio-resistance, metastasis, and tumor invasion (29, 30). The PD-1/PD-L1 signaling plays a crucial function in stemness maintenance of CSCs (31). On the other hand, T cell activity can be suppressed by CSCs (32). A previous study revealed that blockade of the PD-1/PD-L1 pathway with PD-1 orPD-L1 antibodies could inhibit the tumorigenic effect of colon cancer stem cells (33). Therefore, targeting CSCs with anti-PD-1/anti-PD-L1 shows promising therapeutic value. This study revealed that ASCL2 was highly expressed in colon cancer stem cells. In addition, mRNAsi showed higher expression in the ASCL2-high expression group than in the ASCL2-low expression group. Therefore, we inferred that an ASCL2-related cancer stem cell signature was likely to affect the efficacy of colon cancer immunotherapy.

Tumor-infiltrating immune cells (TIICs) are a part of the complex tumor microenvironment (TME). They can be effectively targeted by drugs and are correlated with clinical outcomes (34, 35). Colon adenocarcinoma is infiltrated by various TIICs, including T cells, B cells, NK cells, macrophages, and neutrophils. Previous studies have demonstrated that the density and type of TIICs within COAD affect treatment response and correlate to the prognosistic value (36–38). Several studies reveal that the tumor immune environment influences response to immunotherapeutics (39, 40). A previous study showed that an increased CD8^+^ T cell density in post-treatment serial biopsies from responding melanoma patients treated with pembrolizumab (41). Further, the number of tumor-infiltrating CD4^+^ and CD8^+^ T cells in patients with MSI-H colorectal cancer who benefit from pembrolizumab immunotherapy was significantly higher than in the MSS colorectal cancer (42). Although TIICs and MSI play vital roles in the efficacy of immunotherapy with ICIs, the potential correlation between TIICs and MSI in COAD immunotherapy remains poorly understood. In the present study, ASCL2 expression was significantly correlated with the infiltration levels of CD4+ T cells, CD8+ T cells, B cells, neutrophils, and dendritic cells in various cancer types, including COAD. We revealed potential binding motifs on the promoter region of dual-specificity protein phosphatase 4 (DUSP4) towards transcription factor ASCL2. Moreover, the expression of ASCL2 was negatively associated with DUSP4 in COAD. In other words, ASCL2 could negatively regulate the expression of DUSP4 by binding specifically to the DUSP4 promoter region.

DUSP4 is involved in multiple cellular processes such as cell proliferation and immune response (43). Research shows that DUSP4 is a vital regulator of tumor development. However, it is not clear how DUSP4 affects the clinical and biological effects of tumors. Different tumor types showed different results (44–46). Previous studies revealed that the expression of DUSP4 was negatively associated with distant metastases in colorectal cancer (47). On the other hand, DUSP4 was highly in MSI-H than in MSS tumors (48). In this study, the single-cell RNA sequencing analysis revealed that DUSP4 was highly expressed in CD4^+^ T cells and CD8^+^ T cells. Moreover, DUSP4 expression was significantly correlated with the infiltration levels of CD4^+^ T cells, CD8^+^ T cells, B cells, neutrophils, and dendritic cells in various cancer types. The expression of ASCL2 was significantly correlated with the infiltration levels of CD4+ T cells, CD8+ T cells, B cells, neutrophils, and dendritic cells in various cancer types, including COAD. This implies that ASCL2 can indirectly affect the tumor immune microenvironment by regulating the expression of downstream target gene DUSP4. This revealed that the ASCL2 could affect immunotherapy response by direct regulation of colon cancer stem cells and indirect regulation of tumor-infiltrating immune cells.

The WNT/β-catenin signaling pathway is a tightly controlled pathway that regulates homeostasis and embryogenesis. Dysregulation of the WNT/β-catenin pathway is associated with several cancer types (49, 50). Previous studies revealed that the WNT/β-catenin pathway was aberrantly activated in colon cancer stem cells. Inhibition of the target proteins can block the signaling pathways, thereby affecting the stemness and proliferation of CSCs (51). Meanwhile, a recent study revealed that activation of the WNT/β-catenin pathway in colon cancer stem cells contributes to chemoresistance (52). Furthermore, the WNT/β-catenin pathway might play a vital role in the immunoregulation of the tumor microenvironment (53). Spranger et al. (54) found that overactivation of the WNT/β-catenin pathway reduced infiltration of T cells into tumor-immune microenvironment in a mouse model of primary melanoma, which subsequently decreased the efficacy of the ICIs. In addition, another study reported that overactivation of the WNT/β-catenin pathway could reduce the levels of interferon-γ (IFN-γ), thus suppressing the cytotoxic function of T lymphocytes (55). These findings indicate that the WNT/β-catenin pathway could be involved in immunosuppression and anti-tumor immune responses. Further, this study revealed that ASCL2 and DUSP4 were enriched in the WNT/β-catenin pathway. In addition, ASCL2 was positively correlated with the WNT/β-catenin pathway. However, DUSP4 was negatively regulated with the WNT/β-catenin pathway. Therefore, it was deduced that the immunotherapy-sensitive role of the ASCL2/DUSP4 axis on COAD is partly attributed to the activation of the WNT/β-catenin signaling.

## Conclusion

In conclusion, ASCL2 was highly expressed in COAD. In addition, ASCL2 plays a significant role in microsatellite instability status, cancer stemness, and immune cell infiltration of COAD. Furthermore, the ASCL2/DUSP4 axis was identified as a downstream regulator of COAD. The ASCL2 could be used as a predictor of therapeutic response to anti-PD1/PD-L1 therapy in COAD. However, these results need to be validated in large clinical trials.

## Data Availability Statement

The original contributions presented in the study are included in the article/**Supplementary Material**. Further inquiries can be directed to the corresponding authors.

## Author Contributions

LW and, YZ, and GS conceived and designed the study. LW, SS and XL analyzed the data and revised the images. FQ performed the immunohistochemistry analysis. LW drafted the manuscript. MS and YP revised the manuscript. All authors contributed to the article and approved the submitted version.

## Funding

This work was supported by the Scientific Research Foundation for the Introduced Talents of Jinan Central Hospital (YJRC2021011).

## Conflict of Interest

The authors declare that the research was conducted in the absence of any commercial or financial relationships that could be construed as a potential conflict of interest.

## Publisher’s Note

All claims expressed in this article are solely those of the authors and do not necessarily represent those of their affiliated organizations, or those of the publisher, the editors and the reviewers. Any product that may be evaluated in this article, or claim that may be made by its manufacturer, is not guaranteed or endorsed by the publisher.
