# Supplementary material for: ASCL2 Affects the Efficacy of Immunotherapy in Colon Adenocarcinoma Based on Single-Cell RNA Sequencing Analysis
Source: Front Immunol. 2022 Jun 3;13:829640. doi: 10.3389/fimmu.2022.829640 (PMC9237783; doi:10.3389/fimmu.2022.829640)

Supplementary Figure 1 Identification of DEGs in COAD patients. Heatmap of 1570 DEGs in TCGA-COAD dataset. Red represents up-regulation whereas green represents down-regulation.

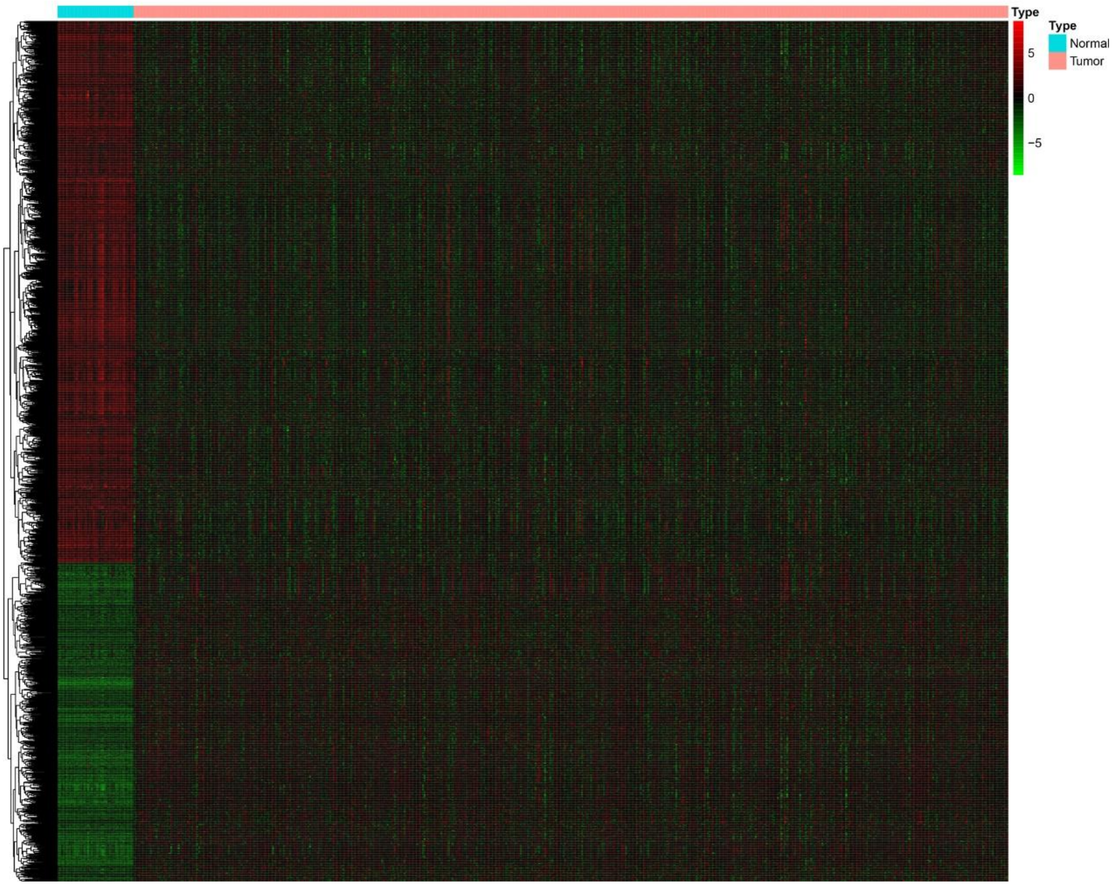

Supplement: Supplementary file 1 [file DataSheet_1.pdf]
