# Supplementary material for: ASCL2 Affects the Efficacy of Immunotherapy in Colon Adenocarcinoma Based on Single-Cell RNA Sequencing Analysis
Source: Front Immunol. 2022 Jun 3;13:829640. doi: 10.3389/fimmu.2022.829640 (PMC9237783; doi:10.3389/fimmu.2022.829640)

Supplementary Figure 2 The results of quality control are presented in Figure A. The number of genes expressed in each cell (nFeature), the number of reads in each cell (nCount), and the percentage of reads mapped to mitochondrial genes (percent.mito). It can be clearly found that mitochondrial genes were all excluded from this analysis. The top 1,000 highly variable genes in the scRNA-Seq data were selected using“vst”of Seurat (Figure B).

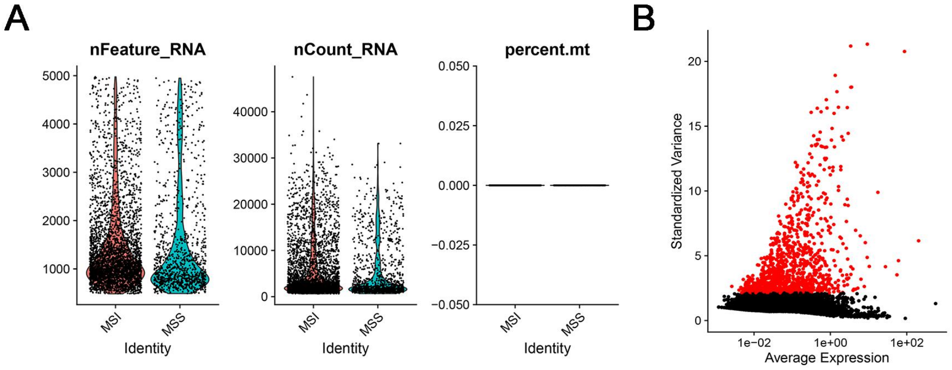

Supplement: Supplementary file 2 [file DataSheet_2.pdf]
