# Supplementary material for: ASCL2 Affects the Efficacy of Immunotherapy in Colon Adenocarcinoma Based on Single-Cell RNA Sequencing Analysis
Source: Front Immunol. 2022 Jun 3;13:829640. doi: 10.3389/fimmu.2022.829640 (PMC9237783; doi:10.3389/fimmu.2022.829640)

Supplementary Figure 3 ASCL2 over-expression and siRNAs plasmids were transfected in colon cancer cells, and found ASCL2 over-expression could promote c-Myc expression (Figure A). Also, c-Myc expression can be suppressed by siRNA-ASCL2 (Figure B).

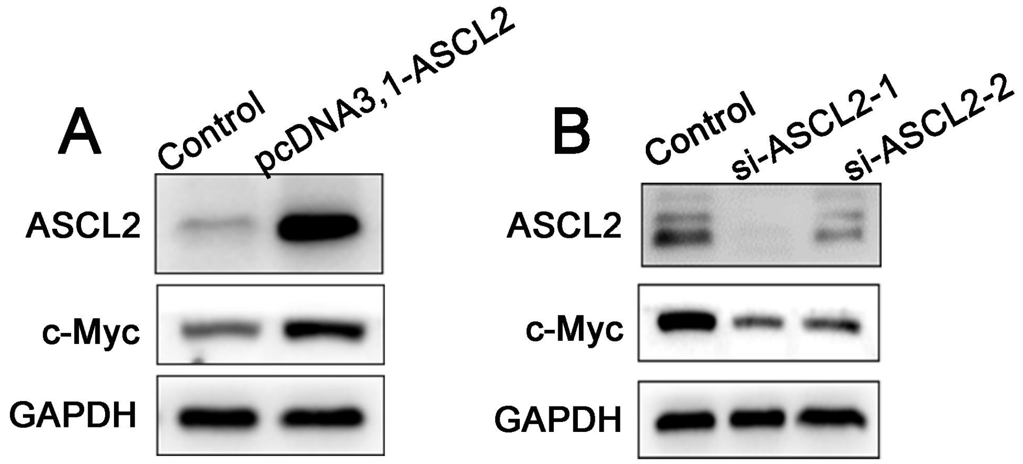

Supplement: Supplementary file 3 [file DataSheet_3.pdf]
