# Supplementary material for: ASCL2 Affects the Efficacy of Immunotherapy in Colon Adenocarcinoma Based on Single-Cell RNA Sequencing Analysis
Source: Front Immunol. 2022 Jun 3;13:829640. doi: 10.3389/fimmu.2022.829640 (PMC9237783; doi:10.3389/fimmu.2022.829640)

As shown in Supplementary Figure 4A and 4B, high mRNA expression of ASCL2 was detected in human colon cancer cell lines and colon cancer tissues via CCLE and TCGA datasets, respectively. Meanwhile, by immunohistochemistry, colon cancer tissues were found to express stronger ASCL2 than normal colon tissues (Supplementary Figure 4C and 4D).

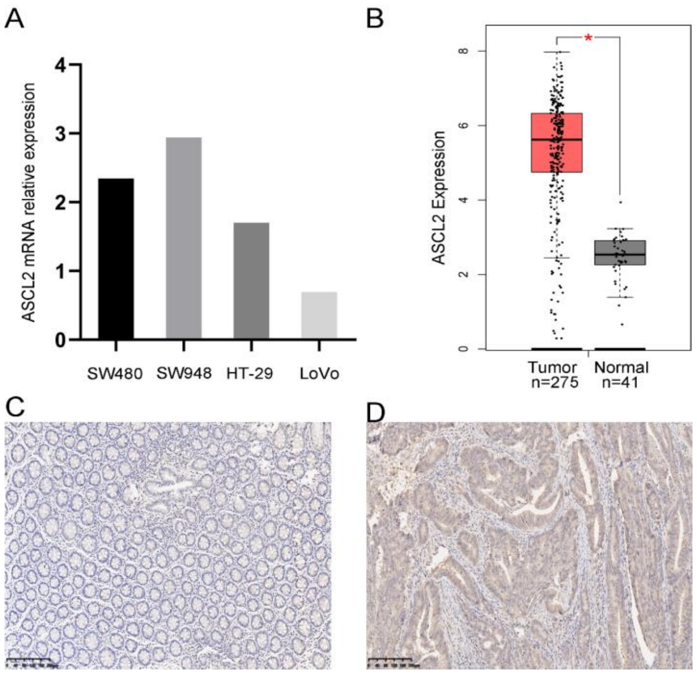

Supplement: Supplementary file 4 [file DataSheet_4.pdf]
