# Supplementary material for: ASCL2 Affects the Efficacy of Immunotherapy in Colon Adenocarcinoma Based on Single-Cell RNA Sequencing Analysis
Source: Front Immunol. 2022 Jun 3;13:829640. doi: 10.3389/fimmu.2022.829640 (PMC9237783; doi:10.3389/fimmu.2022.829640)
Supplement: Supplementary file 5 [file Table_1.pdf]

Supplementary Table 1 Details of specific gene names in blue module

	Gene names
1	ABAT
2	ABCC1
3	ABCC2
4	ABCC4
5	ABHD12
6	ABHD13
7	ACE2
8	ACKR4
9	ACOT8
10	ACSL5
11	ACSL6
12	ADAM10
13	ADRM1
14	AFAP1-AS1
15	AFAP1L2
16	AGPAT4
17	AGT
18	AHCY
19	AHR
20	AIFM1
21	AKAP11
22	AKR7A2
23	ALDH4A1
24	ALOX5
25	AMFR
26	AMT
27	ANKMY2
28	ANO1
29	ANO9
30	ANPEP
31	ANXA1
32	ANXA10
33	ANXA9
34	AOAH
35	APCDD1
36	APMAP
37	APOLD1
38	APOO
39	AREG
40	ARFGAP3
41	ARID3A
42	ARMC2

43	ARNTL2
44	ARSJ
45	AS3MT
46	ASB9
47	ASCL2
48	ASPHD2
49	ASXL1
50	ATP6V1C2
51	ATP7A
52	ATP9A
53	AURKA
54	AX748273
55	AXIN2
56	AZGP1
57	BAK1
58	BCL11A
59	BEX2
60	BHLHB9
61	BLVRB
62	BNIP3L
63	BRCC3
64	BSG
65	C10orf11
66	C12orf57
67	C1orf105
68	C2
69	C6orf123
70	C6orf48
71	C7orf13
72	C7orf31
73	C9orf3
74	CAB39L
75	CABLES2
76	CADPS2
77	CAPN3
78	CARD6
79	CBLN1
80	CBR3
81	CBX4
82	CCDC109B
83	CCDC170
84	CCDC53
85	CCL26
86	CCNDBP1

87 CCPG1
88 CD24
89 CD3EAP
90 CD55
91 CDC42EP1
92 CDCA7
93 CDHR1
94 CDK8
95 CDKN1B
96 CEBPA
97 CEL
98 CELP
99 CFD
100 CFTR
101 CGREF1
102 CHMP4B
103 CHN2
104 CHRM3
105 CHST6
106 CITED1
107 CKMT2
108 CLDN1
109 CLDN15
110 CLDN18
111 CLIC3
112 CLN5
113 CLSTN2
114 CMC4
115 CNTNAP2
116 COCH
117 COG3
118 COL9A3
119 COPG1
120 CPNE1
121 CPNE2
122 CR936796
123 CRELD2
124 CRIM1
125 CRIP1
126 CRYBG3
127 CSE1L
128 CSTF1
129 CTNNBL1
130 CTPS2

131 CTSA
132 CTSE
133 CTSV
134 CTTNBP2
135 CUL4B
136 CXCL14
137 CXCL16
138 CXXC4
139 CXXC5
140 CXorf23
141 CXorf24
142 CYB5B
143 CYB5D1
144 CYB5D2
145 CYP2B6
146 CYP2W1
147 CYP4F2
148 CYP4F3
149 DACH1
150 DAPK1
151 DDB2
152 DDX27
153 DDX5
154 DGAT2
155 DGKA
156 DIAPH2
157 DIDO1
158 DNAJC15
159 DNAJC6
160 DNLZ
161 DNMT3B
162 DNTTIP1
163 DPEP1
164 DPM1
165 DSN1
166 DUS4L
167 DUSP4
168 DUSP6
169 EBPL
170 ECI2
171 EFNA1
172 EIF1AX
173 EIF2S2
174 EIF4E3

175 EIF5A
176 EIF6
177 ELF1
178 ELF5
179 EMILIN3
180 EP300-AS1
181 EPB41L1
182 EPDR1
183 EPHA4
184 EPM2AIP1
185 ERCC5
186 EREG
187 ERP27
188 ETS2
189 ETV4
190 EVA1C
191 EVPL
192 EYA1
193 EZR
194 F10
195 F2RL2
196 F3
197 FAM102B
198 FAM107B
199 FAM114A1
200 FAM122B
201 FAM210B
202 FAM217B
203 FAM228B
204 FAM3B
205 FAM46A
206 FAM57A
207 FAM60A
208 FAM83D
209 FARP1
210 FAS
211 FBXO6
212 FCGRT
213 FDXR
214 FGGY
215 FHL2
216 FITM2
217 FKBP11
218 FLJ32255

219 FLRT3
220 FLVCR1
221 FMR1
222 FOXD1
223 FOXO3
224 FOXQ1
225 FREM1
226 FREM2
227 FUNDC1
228 FUOM
229 FUT8
230 GABRP
231 GALNT1
232 GALNT11
233 GAS2
234 GAS6-AS1
235 GCH1
236 GDF10
237 GDPD5
238 GEMIN4
239 GGH
240 GGT7
241 GJB5
242 GLA
243 GLDC
244 GLOD4
245 GLRX
246 GNA15
247 GNAI1
248 GNG4
249 GOLT1A
250 GPALPP1
251 GPC4
252 GPR115
253 GPR126
254 GPR143
255 GPR160
256 GPR56
257 GPSM2
258 GRB7
259 GRM8
260 GSPT2
261 GSTA4
262 GTF2F2

263 GTF2IRD1
264 GTF3A
265 GYG2
266 GYLTL1B
267 H2AFJ
268 H2AFY2
269 HCG11
270 HENMT1
271 HERC2
272 HILPDA
273 HNRNPL
274 HOXC6
275 HPSE
276 HSD17B10
277 HSPA4L
278 HSPH1
279 HUNK
280 IER5
281 IFT88
282 IGFL2
283 IL15
284 IL18
285 IL1R2
286 INPP1
287 INSM1
288 IRF2
289 IRS2
290 IRX3
291 ISL1
292 JADE3
293 JAK2
294 JPX
295 JUN
296 JUP
297 KAT2B
298 KCNN2
299 KCTD9
300 KDELR3
301 KHDRBS3
302 KIAA0226L
303 KIAA1324L
304 KITLG
305 KLHL23
306 KLK11

307 KPNA3
308 KRBOX4
309 KRT13
310 KRT23
311 KRT40
312 LAGE3
313 LAMP2
314 LAPTM4B
315 LBR
316 LEMD1
317 LGR5
318 LGSN
319 LIG4
320 LINC00525
321 LINC00657
322 LINC00960
323 LINC01003
324 LINC01234
325 LINC01315
326 LINC01420
327 LNX2
328 LOC100288675
329 LOC100505912
330 LOC100506922
331 LOC101060264
332 LOC101927809
333 LOC101928545
334 LOC101928881
335 LOC153684
336 LOC441179
337 LOC729680
338 LOXL1-AS1
339 LPCAT1
340 LPIN1
341 LRFN3
342 LRP11
343 LRRC2
344 LXN
345 LY75
346 LYN
347 LYSMD2
348 LYZ
349 MACC1
350 MAD2L2

351 MAMLD1
352 MAP7D2
353 MAPRE1
354 MARC2
355 MCUR1
356 MEST
357 MICU2
358 MINOS1P1
359 MIPEP
360 MIR31HG
361 MLH1
362 MLPH
363 MOCOS
364 MOCS3
365 MOSPD1
366 MPV17L
367 MRGBP
368 MRPL2
369 MT1E
370 MT1F
371 MT1G
372 MT1H
373 MT1HL1
374 MT1M
375 MT1X
376 MT2A
377 MTA2
378 MTERF1
379 MTTP
380 MYBL2
381 MYC
382 MYEF2
383 N4BP2L2
384 N6AMT2
385 NAA16
386 NAA38
387 NAPG
388 NDRG3
389 NEDD4
390 NEK3
391 NELFCD
392 NEU1
393 NEURL1B
394 NFE2L3

395 NFS1
396 NHLRC3
397 NKD1
398 NKD2
399 NME7
400 NMI
401 NOTUM
402 NOX1
403 NPTX2
404 NR0B2
405 NR1D2
406 NRIP1
407 NRN1
408 NRTN
409 NSDHL
410 NT5C3B
411 NT5E
412 NUCB2
413 NUDT6
414 NUFIP1
415 NUTM2A-AS1
416 NXF3
417 NXT1
418 OR7E37P
419 OSER1
420 OSR2
421 OXCT1
422 PABPC1L
423 PALD1
424 PALM3
425 PAN3
426 PAN3-AS1
427 PARP4
428 PAXIP1-AS1
429 PCDH19
430 PCMTD2
431 PCSK1N
432 PDHA1
433 PDK3
434 PDRG1
435 PHACTR2
436 PHACTR3
437 PHYHIPL
438 PIBF1

439 PIGU
440 PIK3R1
441 PIPOX
442 PKN3
443 PLA2G12B
444 PLA2G2A
445 PLA2G4A
446 PLAG1
447 PLAGL1
448 PLAGL2
449 PLCB4
450 PLEK2
451 PLIN2
452 PLK2
453 PLLP
454 PLOD3
455 PLP2
456 PM20D2
457 PMEPA1
458 PNLIPRP2
459 PNMA2
460 POLA1
461 POLR1D
462 POPDC3
463 POU5F1P3
464 PPAPDC1B
465 PPDPF
466 PPIB
467 PPP1R14C
468 PPP1R3D
469 PRAP1
470 PRDX4
471 PRDX5
472 PRKCQ
473 PROSER1
474 PROSER2
475 PROX1
476 PRPF6
477 PRPS2
478 PRR15
479 PRR9
480 PRSS23
481 PRSS33
482 PSMA7

483 PTGDR
484 PTP4A3
485 PTPRD
486 PTPRO
487 PTPRR
488 PUS7
489 PYGL
490 QPCT
491 QPRT
492 RAB22A
493 RAB27A
494 RAB27B
495 RAB38
496 RALY
497 RAP2C
498 RARRES1
499 RASGEF1A
500 RASL11A
501 RASSF10
502 RBM39
503 RBP2
504 RCN1
505 REEP1
506 RFXAP
507 RGN
508 RHOF
509 RIIAD1
510 RIN2
511 RIPPLY3
512 RLN2
513 RNF113A
514 RNF125
515 RNF128
516 RNF182
517 RNF19B
518 RNF43
519 RNMTL1
520 ROCK2
521 ROMO1
522 RP1-151F17.2
523 RP1-39G22.7
524 RP11-38P22.2
525 RP11-401P9.4
526 RP11-486A14.1

527 RP11-48B3.4
528 RP11-524D16__A.3
529 RP11-5C23.1
530 RP11-75C9.1
531 RPGR
532 RPIA
533 RPS27L
534 RPS6KA3
535 RSPH1
536 S100A16
537 SALL4
538 SAMD12
539 SATB1
540 SATB2
541 SCAND1
542 SCEL
543 SCG5
544 SCML1
545 SCML2
546 SCO2
547 SDC4
548 SDF2L1
549 SDR16C5
550 SEC22B
551 SEMA3C
552 SEMA4B
553 SEMA5A
554 SEMG1
555 SEPHS2
556 SERINC3
557 SERPINA3
558 SERPINB5
559 SESN1
560 SGCB
561 SGMS2
562 SGPP1
563 SHISA9
564 SHROOM2
565 SLC16A14
566 SLC16A7
567 SLC17A9
568 SLC18B1
569 SLC19A3
570 SLC1A7

571 SLC22A3
572 SLC25A15
573 SLC25A43
574 SLC29A1
575 SLC2A12
576 SLC35D3
577 SLC38A11
578 SLC40A1
579 SLC41A2
580 SLC5A6
581 SLC7A6
582 SLC7A8
583 SLC9A8
584 SLFN13
585 SLFN5
586 SLMO2
587 SMAP1
588 SMTNL2
589 SNCAIP
590 SNHG17
591 SNTB1
592 SNX30
593 SOCS2
594 SORL1
595 SOX8
596 SP6
597 SPACA3
598 SPAG4
599 SPATA18
600 SPATA2
601 SPATC1L
602 SPIN3
603 SPIRE1
604 SPPL2A
605 SPRED1
606 SPRR1A
607 SRPX2
608 SS18L1
609 SSUH2
610 ST3GAL4-AS1
611 ST6GAL1
612 ST6GALNAC2
613 STAG2
614 STC2

615 STRN3
616 STX16
617 SUCLA2
618 SYAP1
619 SYNE4
620 SYTL1
621 TAX1BP1
622 TBC1D4
623 TBCK
624 TCEAL1
625 TCFL5
626 TFAP2A
627 TFF2
628 TGFBI
629 TGIF2
630 THOC2
631 TIPARP
632 TLE2
633 TLR3
634 TM4SF4
635 TM9SF4
636 TMEM106B
637 TMEM117
638 TMEM150C
639 TMEM163
640 TMEM170B
641 TMEM176A
642 TMEM187
643 TMEM192
644 TMEM220
645 TMEM243
646 TMEM252
647 TMEM256
648 TMEM260
649 TMEM263
650 TMEM64
651 TMEM9
652 TMEM98
653 TMTC4
654 TNFAIP8
655 TNFSF10
656 TNFSF9
657 TNIK
658 TNMD

659 TNNC2
660 TNNT1
661 TOMM34
662 TP53
663 TP53RK
664 TPBG
665 TPRG1
666 TPX2
667 TRAPPC6A
668 TRAPPC6B
669 TRIB2
670 TRIM13
671 TRIM16
672 TRIM7
673 TRMT2B
674 TRNP1
675 TSPAN33
676 TSPAN6
677 TTC32
678 TTC9
679 TTI1
680 TTPAL
681 TUBB2A
682 TUFT1
683 TVP23B
684 TXLNG
685 UAP1L1
686 UBE2C
687 UBE2M
688 UBL3
689 UCHL3
690 UFM1
691 ULBP2
692 UMODL1
693 UPP1
694 UPRT
695 USF1
696 USP9X
697 VANGL2
698 VAV3
699 VBP1
700 VIL1
701 VMP1
702 VNN1

703 WDR35
704 WDR54
705 WDR77
706 WFDC21P
707 WIF1
708 WNT11
709 XPNPEP2
710 Y16709
711 YAE1D1
712 YTHDF1
713 YWHAE
714 ZAK
715 ZBED3
716 ZBED6CL
717 ZBTB10
718 ZBTB38
719 ZBTB8A
720 ZDHHC9
721 ZFAS1
722 ZGPAT
723 ZIC2
724 ZNF204P
725 ZNF217
726 ZNF232
727 ZNF275
728 ZNF280C
729 ZNF426
730 ZNF43
731 ZNF439
732 ZNF652
733 ZNF813
734 ZNF818P
735 ZNRF3
736 ZXDA
