# Supplementary material for: ASCL2 Affects the Efficacy of Immunotherapy in Colon Adenocarcinoma Based on Single-Cell RNA Sequencing Analysis
Source: Front Immunol. 2022 Jun 3;13:829640. doi: 10.3389/fimmu.2022.829640 (PMC9237783; doi:10.3389/fimmu.2022.829640)
Supplement: Supplementary file 6 [file Table_2.pdf]

id	logFC	AveExpr	t	P.Value	adj.P.Val	B
Y16709	-2.19162	11.489037	-32.90641	3.68E-129	7.98E-125	282.31104
HNRNPL	1.7700902	8.1814019	21.486149	9.46E-74	1.02E-69	156.80199
SEC22B	-1.375233	9.3183729	-21.00502	2.27E-71	1.64E-67	151.39277
MTA2	1.2204168	4.7476654	20.894532	7.99E-71	4.33E-67	150.15166
HPSE	1.472753	5.1879855	19.541021	3.70E-64	1.34E-60	134.99991
STRN3	1.2206629	5.9024394	19.46139	9.10E-64	2.81E-60	134.11237
MLH1	-1.328848	8.0095828	-19.29038	6.26E-63	1.69E-59	132.20819
RASL11A	1.3506723	4.8832289	18.885254	5.97E-61	1.32E-57	127.70784
H2AFJ	-1.086158	7.5490893	-16.98815	8.42E-52	1.66E-48	106.90034
SAMD12	-1.134641	5.1787786	-16.6203	4.69E-50	7.80E-47	102.93095
RNF19B	1.0722374	7.6520289	15.197257	2.02E-43	2.19E-40	87.841611
PRPF39	1.1673908	6.7031433	14.697302	3.84E-41	3.62E-38	82.658078
TRIM7	1.0265495	4.3858668	14.62521	8.15E-41	7.06E-38	81.916359
SRSF6	1.2670419	8.3171077	14.343807	1.51E-39	1.21E-36	79.035687
ZNF514	-1.250475	6.3827257	-14.29023	2.62E-39	2.02E-36	78.489899
EPDR1	-2.009808	7.9445093	-13.71737	9.11E-37	5.80E-34	72.711066
TSPAN6	-1.26155	10.188327	-13.52282	6.49E-36	3.90E-33	70.773168
KLHL23	-1.035708	6.9715061	-13.41126	1.99E-35	1.16E-32	69.66777
SEMA5A	-1.23314	7.1585567	-13.36485	3.16E-35	1.80E-32	69.209323
ATP9A	-1.160135	7.8363588	-13.33265	4.36E-35	2.37E-32	68.891646
RPL22L1	1.4734074	9.4468069	13.271012	8.07E-35	3.97E-32	68.28453
EP300-AS1	1.2703295	3.768138	13.250929	9.86E-35	4.74E-32	68.087026
HSPH1	-1.042022	8.257415	-13.145	2.83E-34	1.33E-31	67.047705
ASPHD2	1.0507894	5.7640994	12.982806	1.41E-33	6.34E-31	65.464466
GNLY	1.4075697	5.3780356	12.911396	2.84E-33	1.23E-30	64.77054
QPRT	-1.902191	7.5600501	-12.5834	6.99E-32	2.48E-29	61.608627
HOXC6	2.5661355	5.557742	12.548339	9.82E-32	3.38E-29	61.273185
WARS	1.1165708	8.8917353	12.542172	1.04E-31	3.47E-29	61.21423
AXIN2	-1.180956	6.894209	-12.52665	1.21E-31	3.98E-29	61.065917
EIF5A	1.4994563	8.9102349	12.477804	1.94E-31	6.28E-29	60.599817
AMFR	1.0621227	6.2263619	12.36811	5.60E-31	1.68E-28	59.556651
FBXO6	1.2223284	6.8874025	12.106345	6.84E-30	1.83E-27	57.087646
LINC01003	-1.229245	7.4250248	-12.07531	9.18E-30	2.40E-27	56.79683
FTX	-1.302512	6.1629279	-11.92823	3.69E-29	8.88E-27	55.424468
RNF43	-1.429873	9.3220859	-11.92415	3.84E-29	9.13E-27	55.386481
VAV3	-1.969482	7.4769832	-11.86337	6.80E-29	1.55E-26	54.822237
CAB39L	-1.308607	6.1962875	-11.63194	5.91E-28	1.20E-25	52.688899
LAPTM4B	-1.436376	9.8380912	-11.60775	7.40E-28	1.48E-25	52.467323
HSPA4L	1.5573759	4.5399471	11.605543	7.56E-28	1.50E-25	52.44716
USP18	1.154841	5.7293761	11.472165	2.60E-27	4.89E-25	51.23045
MACC1	-1.248745	6.7050544	-11.43005	3.83E-27	7.08E-25	50.847961
VMP1	-1.350481	8.4999639	-11.41972	4.21E-27	7.72E-25	50.754332
TGFBI	-1.090317	11.637463	-11.31537	1.10E-26	1.95E-24	49.810832
SECTM1	1.1990295	7.0613531	11.30931	1.16E-26	2.04E-24	49.756174
KRT23	-2.847396	7.2388617	-11.21126	2.83E-26	4.65E-24	48.874664
PLCB4	-2.106752	8.5882453	-11.19667	3.24E-26	5.27E-24	48.743928
DACH1	-1.685899	6.7090299	-11.09711	7.98E-26	1.23E-23	47.854334
ULBP2	1.0047396	5.2006064	11.007504	1.79E-25	2.58E-23	47.05784
KIAA0226L	-2.155323	8.4238524	-10.9968	1.97E-25	2.82E-23	46.96292
A1CF	-1.416574	6.6590036	-10.94943	3.02E-25	4.24E-23	46.543799
CXCL14	-1.670832	7.657251	-10.94076	3.26E-25	4.53E-23	46.467186
NOX1	-1.801608	8.640988	-10.93003	3.59E-25	4.95E-23	46.372436
ENO2	1.2900284	6.0934104	10.859869	6.72E-25	8.88E-23	45.754315
CCDC68	1.1023203	6.2104961	10.857226	6.88E-25	9.03E-23	45.731081
GPSM2	-1.033405	7.6285455	-10.80845	1.06E-24	1.38E-22	45.302945
TRIB2	1.0683549	6.1747881	10.74699	1.83E-24	2.30E-22	44.765117
CTTNBP2	-1.27305	5.489789	-10.73079	2.12E-24	2.59E-22	44.623671
SEMG1	1.3727096	3.442182	10.67056	3.61E-24	4.20E-22	44.099046
PRR15	-1.121655	8.8337978	-10.60413	6.48E-24	7.34E-22	43.522586
SEMA3C	-1.06926	7.1580061	-10.60215	6.59E-24	7.43E-22	43.505427
SESN1	-1.289847	8.1463539	-10.55703	9.80E-24	1.08E-21	43.115233
HENMT1	-1.119406	7.422747	-10.42663	3.06E-23	3.20E-21	41.993668
TMEM176A	-1.161538	10.119919	-10.39247	4.12E-23	4.29E-21	41.701357
IFI6	1.6244449	8.11279	10.345021	6.21E-23	6.35E-21	41.296382
DUSP4	1.532766	6.1856456	10.326959	7.26E-23	7.32E-21	41.142542
PMEPA1	-1.207098	8.4903542	-10.25529	1.35E-22	1.27E-20	40.533797
MUC20	-1.003952	6.8497349	-10.13012	3.94E-22	3.50E-20	39.477431
PRAP1	-1.510202	7.9681797	-10.1186	4.35E-22	3.83E-20	39.38065
TNFSF9	1.0489601	5.0964801	10.065052	6.86E-22	5.81E-20	38.931745
RARRES3	1.4318916	7.5495973	10.000748	1.18E-21	9.72E-20	38.394752
PLK2	1.1731494	6.864309	9.9056346	2.64E-21	2.12E-19	37.604736
GPR143	-1.247443	5.6439267	-9.84843	4.27E-21	3.18E-19	37.132069
LY75	-1.175888	8.4566989	-9.737031	1.08E-20	7.50E-19	36.216975
GZMA	1.4209356	5.8791613	9.7309453	1.14E-20	7.81E-19	36.167193
PPP1R14D	-1.289572	7.8136365	-9.720624	1.24E-20	8.49E-19	36.082804
RAMP1	1.23169	6.67521	9.654518	2.15E-20	1.41E-18	35.5438
GALNT6	-1.04063	7.4987467	-9.610275	3.10E-20	1.97E-18	35.184483
HCAR3	1.7487641	5.4954522	9.5521982	4.99E-20	3.09E-18	34.714557
ID1	-1.480739	10.561225	-9.502611	7.50E-20	4.47E-18	34.314902
C10orf99	-2.269256	8.6799076	-9.398156	1.76E-19	9.88E-18	33.477815
SYTL1	1.0587583	6.4481547	9.3597375	2.40E-19	1.32E-17	33.171582
SLC7A11	1.0340946	6.2192739	9.3113654	3.55E-19	1.92E-17	32.787273
CHAC2	1.0198915	6.1653965	9.2750884	4.75E-19	2.53E-17	32.499988
MICB	1.1044629	6.2386247	9.1820873	1.00E-18	5.08E-17	31.767158
MT1X	1.101402	9.1658323	9.1794491	1.02E-18	5.18E-17	31.746446
GPR160	-1.143456	9.9047759	-9.113086	1.74E-18	8.43E-17	31.226874
SLC1A1	1.0112	6.4674715	9.099205	1.94E-18	9.33E-17	31.118538
OSR2	1.1109108	4.6976283	9.0341518	3.25E-18	1.51E-16	30.612425
FLJ22763	-1.359403	4.9186115	-9.009478	3.94E-18	1.81E-16	30.421156
CCL4	1.0836954	7.2036888	9.0091416	3.95E-18	1.81E-16	30.418548
QPCT	-1.465896	7.096494	-9.003588	4.13E-18	1.88E-16	30.375551
ASCL2	-1.133068	6.3455209	-8.959621	5.84E-18	2.60E-16	30.035823
IDO1	1.3316926	6.5592594	8.9452949	6.53E-18	2.89E-16	29.925388
CCDC58	1.0614719	6.5163551	8.9213246	7.88E-18	3.46E-16	29.740901
HLA-DMA	1.1563976	8.0743763	8.8008799	2.02E-17	8.39E-16	28.819403
CXCL13	1.894651	5.339627	8.7586332	2.80E-17	1.13E-15	28.498372
KISS1R	1.1006921	4.4393617	8.7557872	2.86E-17	1.15E-15	28.476786
GNG4	-1.125117	5.2204139	-8.724225	3.65E-17	1.44E-15	28.237751
PLLP	1.1641103	6.5235872	8.7212021	3.73E-17	1.47E-15	28.21489
GGH	-1.105419	9.6916322	-8.705477	4.21E-17	1.64E-15	28.096065
AGR2	1.2120447	10.441059	8.656729	6.12E-17	2.33E-15	27.728725
BCL2A1	1.3794987	5.9306833	8.5519063	1.36E-16	4.90E-15	26.944057
CCL8	1.296628	5.9930169	8.5071608	1.91E-16	6.74E-15	26.611293
CXCL10	1.5546513	7.456864	8.476655	2.41E-16	8.33E-15	26.385181
FCGR3B	1.0751915	5.9305486	8.4448278	3.06E-16	1.04E-14	26.149928
APCDD1	-1.619294	8.3352187	-8.428296	3.47E-16	1.17E-14	26.027997
PPP1R14C	-1.292762	6.8851582	-8.383524	4.85E-16	1.60E-14	25.698691
TNFSF13B	1.0708332	6.5077696	8.379347	5.01E-16	1.64E-14	25.668033
CCL5	1.080258	6.9273312	8.2752981	1.09E-15	3.38E-14	24.90818
IFIT3	1.0428242	7.1124054	8.264107	1.18E-15	3.64E-14	24.826885
ACE2	-1.721975	6.766013	-8.231205	1.51E-15	4.57E-14	24.588364
CRIP1	1.1757209	8.6571285	8.1772939	2.24E-15	6.58E-14	24.199124
RP11-44F2	-1.021403	5.3393342	-8.074134	4.76E-15	1.33E-13	23.459807
FREM2	-1.142127	3.6101235	-8.031593	6.49E-15	1.79E-13	23.157045
MT1E	1.0495373	9.3220069	7.8966539	1.72E-14	4.34E-13	22.204944
SPINK1	-1.652139	10.865458	-7.892749	1.76E-14	4.44E-13	22.177583
BST2	1.1449124	7.8048913	7.8599113	2.23E-14	5.49E-13	21.947885
NCF2	1.0331754	5.7688189	7.8532825	2.34E-14	5.74E-13	21.901609
MMP1	2.0089566	8.723915	7.8091689	3.20E-14	7.76E-13	21.594431
PBK	1.0128951	7.9566915	7.8016135	3.38E-14	8.14E-13	21.541957
SLC16A14	1.0944163	5.6591348	7.733822	5.46E-14	1.27E-12	21.072924
CDHR1	-1.143806	5.6893322	-7.60808	1.32E-13	2.91E-12	20.21155
COL9A3	-1.185165	6.1257292	-7.555009	1.90E-13	4.09E-12	19.851381
CXCL9	1.4540811	7.1393049	7.549152	1.98E-13	4.26E-12	19.811752
CELP	-1.355115	6.1057522	-7.537067	2.15E-13	4.60E-12	19.730069
GUCY2C	-1.09576	8.686349	-7.534911	2.19E-13	4.66E-12	19.715511
HMGCS2	-1.331133	6.9198584	-7.522997	2.37E-13	5.03E-12	19.635103
TMEM45A	1.1436758	6.4078801	7.4260232	4.62E-13	9.17E-12	18.984436
CEACAM6	-1.040794	12.041613	-7.380537	6.30E-13	1.22E-11	18.681582
CXCL8	1.4478586	8.6291352	7.3778411	6.41E-13	1.24E-11	18.663682
DPEP1	-1.472284	7.9058487	-7.334971	8.58E-13	1.63E-11	18.379709
FOXD1	1.1151272	4.7394893	7.2917778	1.15E-12	2.13E-11	18.094955
MEP1A	-1.493626	8.4136888	-7.290283	1.16E-12	2.15E-11	18.085126
CXCL11	1.6890613	5.2799775	7.2231031	1.82E-12	3.26E-11	17.645031
REEP1	-1.20767	5.7113194	-7.180469	2.42E-12	4.26E-11	17.367459
SPP1	1.1515698	5.8857928	7.1758655	2.49E-12	4.38E-11	17.337569
TNFAIP6	1.1644657	5.8718068	7.0549751	5.54E-12	9.27E-11	16.558252
CPE	-1.163048	6.6913528	-7.03551	6.30E-12	1.04E-10	16.43379
FABP1	-1.406226	8.3162694	-6.998221	8.03E-12	1.30E-10	16.196148
PCP4	-1.399838	5.1928757	-6.944375	1.14E-11	1.80E-10	15.85484
CCL18	1.2861905	7.7171486	6.8753891	1.78E-11	2.71E-10	15.420755
FOXQ1	-1.174666	9.5881117	-6.866277	1.89E-11	2.85E-10	15.36369
SELENBP1	-1.098468	9.2746157	-6.830409	2.37E-11	3.53E-10	15.139667
ERP27	-1.245528	6.0360768	-6.822493	2.50E-11	3.70E-10	15.09036
IL1B	1.0048834	7.3977108	6.7727082	3.43E-11	4.97E-10	14.78134
REG4	1.963915	7.2642413	6.7524608	3.90E-11	5.60E-10	14.656204
MMP12	1.1052558	9.9445594	6.7270531	4.58E-11	6.47E-10	14.499621
ISX	-1.21887	6.5245698	-6.584834	1.12E-10	1.47E-09	13.632315
KRT20	-1.39562	10.226406	-6.479909	2.13E-10	2.70E-09	13.002489
CEL	-1.837499	6.8668907	-6.347486	4.77E-10	5.70E-09	12.219884
TFF2	1.0643464	5.8859845	6.332567	5.22E-10	6.18E-09	12.132581
MMP7	-1.299328	8.0629403	-6.2922	6.66E-10	7.79E-09	11.89724
EREG	-1.146281	6.1325388	-6.235984	9.31E-10	1.06E-08	11.571647
CXCL5	1.0517508	4.5884007	6.2138092	1.06E-09	1.19E-08	11.443908
PLA2G2A	1.6983361	9.455753	6.1893515	1.23E-09	1.36E-08	11.30347
SDR16C5	1.3721695	6.6058475	6.0194027	3.31E-09	3.37E-08	10.340842
MUC12	-1.228563	6.9457173	-6.005271	3.59E-09	3.64E-08	10.26184
SLC35D3	-1.413729	5.3510935	-5.998313	3.74E-09	3.77E-08	10.223007
PTGS2	1.1099868	5.4830101	5.8826606	7.24E-09	6.92E-08	9.5832235
ZIC2	1.3709734	4.672906	5.6562709	2.56E-08	2.21E-07	8.3624827
PLA2G4A	1.1729357	5.4812847	5.652065	2.62E-08	2.26E-07	8.3402034
LEFTY1	-1.692174	8.1457051	-5.582147	3.83E-08	3.20E-07	7.9719736
MMP3	1.3652396	8.2929125	5.4786954	6.69E-08	5.36E-07	7.4346014
CA2	1.2975274	7.929793	5.4358761	8.40E-08	6.59E-07	7.2147959
DEFA6	-1.421796	7.1678184	-5.281212	1.89E-07	1.39E-06	6.4336945
IFI44L	1.0786208	5.8317279	5.2324122	2.43E-07	1.74E-06	6.1914391
AGR3	1.3880662	8.7080753	5.2111241	2.71E-07	1.93E-06	6.0863916
PRAC1	-1.910585	6.9845033	-5.175817	3.25E-07	2.27E-06	5.9130174
OLFM4	-1.895995	10.236107	-5.129857	4.10E-07	2.81E-06	5.6889243
CTSE	1.1609891	6.5076295	5.105343	4.64E-07	3.13E-06	5.5701349
SLC26A3	-1.275554	6.2776374	-5.07445	5.42E-07	3.61E-06	5.4211703
WIF1	-1.004157	3.73827	-4.968368	9.18E-07	5.77E-06	4.9158799
TAC1	-1.025164	4.00815	-4.915126	1.19E-06	7.32E-06	4.6659329
PLAC8	1.087338	9.1002237	4.7865631	2.22E-06	1.28E-05	4.0725142
CALB1	1.0661914	3.7343162	4.5744628	5.98E-06	3.14E-05	3.1250429
DEFA5	-1.437626	6.0847318	-4.371107	1.49E-05	7.22E-05	2.253868
REG1A	1.4478548	7.9424734	3.9308725	9.61E-05	0.0003771	0.4945981
REG1B	1.3370509	6.5446095	3.8859651	0.0001151	0.0004435	0.324989
MUC2	1.0907927	8.8255999	3.8316208	0.0001429	0.0005391	0.122197
