# Supplementary material for: ASCL2 Affects the Efficacy of Immunotherapy in Colon Adenocarcinoma Based on Single-Cell RNA Sequencing Analysis
Source: Front Immunol. 2022 Jun 3;13:829640. doi: 10.3389/fimmu.2022.829640 (PMC9237783; doi:10.3389/fimmu.2022.829640)
Supplement: Supplementary file 7 [file Table_3.pdf]

Supplementary Table 3 Top 10 in PPI network ranked by DMNC method

Rank	Name	Score
1	HOXC6	0.4634631
1	APCDD1	0.4634631
1	ASCL2	0.4634631
4	ZIC2	0.4537838
5	RNF43	0.4279425
6	FOXD1	0.3803933
7	OSR2	0.3803933
8	MLH1	0.3328441
9	AMFR	0.3241313
10	WIF1	0.3207301
