# Supplementary material for: ASCL2 Affects the Efficacy of Immunotherapy in Colon Adenocarcinoma Based on Single-Cell RNA Sequencing Analysis
Source: Front Immunol. 2022 Jun 3;13:829640. doi: 10.3389/fimmu.2022.829640 (PMC9237783; doi:10.3389/fimmu.2022.829640)
Supplement: Supplementary file 8 [file Table_4.pdf]

id	logFC	AveExpr	t	P.Value	adj.P.Val	B
CDH3	6.7248437	11.144624	42.435365	4.54E-170	8.87E-166	377.59572
KRT80	7.0829068	9.7156114	37.105118	1.77E-147	1.73E-143	326.04313
ESM1	5.8648333	7.4379113	36.107317	4.45E-143	2.90E-139	315.99096
ETV4	5.619806	11.415567	32.914008	1.22E-128	5.96E-125	282.99012
AJUBA	3.3677898	9.5775185	31.708776	4.67E-123	1.73E-119	270.2183
WNT2	5.9677479	7.7119589	31.696765	5.32E-123	1.73E-119	270.09018
MTHFD1L	2.5776694	10.715671	30.945083	1.75E-119	4.90E-116	262.04066
OTOP3	-5.372413	0.9228302	-30.2673	2.75E-116	6.72E-113	254.73009
KRT24	-5.834598	0.9281451	-29.3649	5.30E-112	1.15E-108	244.92331
OTOP2	-9.621961	2.5626435	-29.1941	3.46E-111	6.77E-108	243.05808
CLDN1	5.0336697	11.186543	26.965619	1.88E-100	3.34E-97	218.48175
CEMIP	5.3062531	11.647573	26.522305	2.69E-98	4.38E-95	213.54526
LRP8	3.4722222	9.4591041	26.391442	1.17E-97	1.76E-94	212.08542
DHRS7C	-3.240029	0.6244614	-26.26633	4.75E-97	6.64E-94	210.68863
LGI1	-4.918797	1.298275	-25.26151	3.89E-92	5.07E-89	199.4362
BEST4	-6.282819	5.6228057	-24.92091	1.82E-90	2.23E-87	195.60966
INHBA	5.5745827	9.6165067	24.47889	2.70E-88	3.11E-85	190.63595
CA7	-6.846107	5.5442947	-24.41662	5.47E-88	5.95E-85	189.93461
TRIB3	3.8634213	11.060719	23.97113	8.51E-86	8.11E-83	184.91343
FOXQ1	6.3269595	9.651208	23.969188	8.70E-86	8.11E-83	184.89152
TRIP13	2.6092159	9.7979588	23.890653	2.12E-85	1.89E-82	184.0057
NFE2L3	2.6444472	12.0468	23.846885	3.48E-85	2.96E-82	183.51194
PRSS22	4.7602522	9.1533222	23.674663	2.46E-84	2.00E-81	181.56862
GRIN2D	5.1116569	9.8247641	23.571376	7.93E-84	6.20E-81	180.40279
EPOP	3.918963	8.5405881	23.215166	4.52E-82	3.40E-79	176.38059
CHAT	-3.402507	0.7264371	-23.06755	2.41E-81	1.75E-78	174.71318
CLEC3B	-4.420982	6.4671817	-22.88036	2.02E-80	1.41E-77	172.59843
PCSK2	-5.708079	2.020534	-22.8387	3.25E-80	2.19E-77	172.12779
SLC25A34	-4.102796	5.4843923	-22.52596	1.13E-78	7.38E-76	168.59408
GCNT2	-4.149135	5.9414619	-22.4805	1.90E-78	1.20E-75	168.08042
EPHX4	4.7470589	7.4823745	22.450467	2.67E-78	1.63E-75	167.74103
SLCO4A1	3.4498635	10.982783	22.364765	7.06E-78	4.19E-75	166.77269
MAS1L	-2.564252	0.5669945	-22.34418	8.92E-78	5.13E-75	166.54011
MMP27	-2.638892	0.5769724	-21.97914	5.63E-76	3.06E-73	162.41603
CMTM5	-3.578967	1.1124659	-21.97212	6.10E-76	3.22E-73	162.33681
FJX1	3.2618278	7.1631047	21.92663	1.02E-75	5.26E-73	161.82297
ABCG2	-4.799507	6.4951767	-21.9178	1.13E-75	5.67E-73	161.72326
DAO	-5.00548	1.7341937	-21.87351	1.87E-75	9.13E-73	161.22304
RERGL	-5.33273	1.9541777	-21.85781	2.23E-75	1.06E-72	161.04573
MYOC	-6.120433	1.783768	-21.80877	3.89E-75	1.81E-72	160.49193
FUT1	3.1722395	8.040142	21.734539	9.04E-75	4.11E-72	159.65365
ACAN	4.0307286	7.9280245	21.675548	1.77E-74	7.85E-72	158.98758
FAM107A	-3.713633	6.5097734	-21.33566	8.34E-73	3.47E-70	155.15156
SLC17A8	-4.505142	1.3303306	-21.31311	1.08E-72	4.39E-70	154.8971
MDFI	3.9813582	8.5545909	21.30509	1.18E-72	4.71E-70	154.80664
IQANK1	3.0039417	10.032719	21.217798	3.17E-72	1.22E-69	153.82204
PHLPP2	-2.438735	9.5097775	-21.20778	3.56E-72	1.34E-69	153.70904
FAM180B	-3.407575	1.0575047	-21.20584	3.63E-72	1.34E-69	153.68714
ENC1	2.0619021	12.485976	21.189411	4.38E-72	1.59E-69	153.50191
NGB	-3.866827	1.0161239	-21.07802	1.55E-71	5.50E-69	152.24589
DUSP14	2.1583533	9.0187351	20.924486	8.81E-71	3.08E-68	150.51558
BLACAT1	5.3352152	6.8176595	20.900858	1.15E-70	3.95E-68	150.24936
IL6R	-2.864951	8.9029933	-20.86477	1.73E-70	5.84E-68	149.84284
SALL4	4.58216	6.3260782	20.84977	2.05E-70	6.80E-68	149.67384
LYVE1	-4.496916	6.3866248	-20.78564	4.24E-70	1.36E-67	148.95151
COL11A1	6.1251329	9.1654533	20.752804	6.15E-70	1.94E-67	148.5818
KHDRBS2	-3.491902	1.0593665	-20.74978	6.36E-70	1.98E-67	148.54775
SPTBN2	3.2769073	10.147077	20.667048	1.62E-69	4.96E-67	147.61637
CDH10	-2.63858	0.6698652	-20.62464	2.62E-69	7.89E-67	147.13907
KIAA1549	2.4973471	9.7855352	20.602217	3.38E-69	1.00E-66	146.88675
CPNE7	5.4859201	9.0638569	20.577794	4.45E-69	1.30E-66	146.61193
VSTM2A	-5.761559	2.2242395	-20.55058	6.06E-69	1.74E-66	146.3057
SGCG	-3.483143	1.234947	-20.47454	1.43E-68	4.06E-66	145.45048
AADACL2	-3.085427	0.635157	-20.43565	2.22E-68	6.12E-66	145.01309
SIM2	4.5725862	8.44614	20.332085	7.16E-68	1.94E-65	143.84893
ORC6	2.3544767	9.0290884	20.201899	3.11E-67	8.23E-65	142.38638
SPIB	-5.508347	5.9198858	-20.20031	3.17E-67	8.26E-65	142.36849
CBX2	3.8027231	8.6355306	20.142076	6.11E-67	1.55E-64	141.71465
STMN4	-3.794563	1.3950114	-20.06135	1.52E-66	3.76E-64	140.8086
PHOX2B	-4.521035	1.5237496	-20.06041	1.54E-66	3.76E-64	140.79802
TTC26	2.0882421	8.312258	20.047966	1.77E-66	4.27E-64	140.65839
USP2	-4.098141	6.381887	-19.99981	3.04E-66	7.26E-64	140.11818
NPY2R	-4.125347	1.2190296	-19.95364	5.12E-66	1.21E-63	139.60029
SCGN	-5.950818	3.6873738	-19.90251	9.11E-66	2.12E-63	139.02701
TNFRSF12A	2.7494833	10.721936	19.900602	9.30E-66	2.14E-63	139.00561
SPATA12	3.5346434	5.0440506	19.887523	1.08E-65	2.45E-63	138.85899
TEAD4	2.4243897	9.8329205	19.737768	5.82E-65	1.29E-62	137.18111
C2orf88	-3.892965	8.2782089	-19.68387	1.07E-64	2.35E-62	136.57769
MMP7	7.1942076	9.3750592	19.650469	1.55E-64	3.38E-62	136.20378
ZC3HAV1L	2.5357352	7.9755333	19.636116	1.83E-64	3.93E-62	136.04315
CDKN2B	-3.350993	9.1116789	-19.61073	2.43E-64	5.13E-62	135.75906
KRTAP13-2	-4.011015	0.8218093	-19.6104	2.44E-64	5.13E-62	135.75545
DDN	4.5716056	6.2560417	19.60233	2.67E-64	5.56E-62	135.66511
SEC14L2	2.6273499	7.9687544	19.580123	3.43E-64	7.06E-62	135.41668
PLEKHN1	3.6423767	6.8829663	19.55632	4.48E-64	9.13E-62	135.15043
PDX1	4.9841038	8.5717468	19.539053	5.44E-64	1.10E-61	134.95733
GUCA2B	-8.141941	4.1805423	-19.49737	8.69E-64	1.74E-61	134.49128
PPM1H	2.3229795	10.535304	19.427094	1.91E-63	3.78E-61	133.70583
PLP1	-6.20598	3.8689386	-19.38553	3.05E-63	5.97E-61	133.24146
GTF2IRD1	2.1144452	11.20324	19.3577	4.17E-63	8.08E-61	132.93067
CFAP92	3.6868152	6.9999488	19.304902	7.55E-63	1.45E-60	132.34117
ABCA8	-5.550468	5.3578632	-19.23199	1.71E-62	3.25E-60	131.5275
TMIGD1	-8.570735	4.0127296	-19.22774	1.79E-62	3.37E-60	131.4801
PYY	-7.752314	4.0045661	-19.16117	3.78E-62	6.98E-60	130.7377
FAM135B	-4.448477	2.0854643	-19.12565	5.63E-62	1.03E-59	130.3417
SLC7A5	2.9654867	12.417254	19.067383	1.08E-61	1.96E-59	129.69243
APLN	3.5579106	7.9254361	19.024035	1.76E-61	3.15E-59	129.20961
MAMDC2	-5.559076	4.6433012	-18.92795	5.15E-61	9.07E-59	128.14006
XRCC2	2.5185823	8.1608165	18.904955	6.66E-61	1.16E-58	127.88423
CGREF1	3.8618421	9.3904597	18.879829	8.82E-61	1.53E-58	127.60477
AUNIP	2.6092596	7.4323452	18.863967	1.05E-60	1.81E-58	127.42837
PKIB	-4.35718	8.1852846	-18.82662	1.60E-60	2.72E-58	127.0132
EGFL6	3.7421143	6.499238	18.819449	1.73E-60	2.92E-58	126.93344
CADM3	-5.793384	4.3491301	-18.81449	1.83E-60	3.06E-58	126.87832
PLAC9	-2.512909	6.0646353	-18.80229	2.10E-60	3.48E-58	126.74279
EVA1A	3.9464747	7.8133194	18.78904	2.43E-60	4.00E-58	126.5955
SLC30A10	-6.433831	2.9948908	-18.77031	3.00E-60	4.89E-58	126.38737
ASPA	-3.950641	4.005061	-18.76952	3.02E-60	4.89E-58	126.37859
TMEFF2	-3.665346	1.4284146	-18.63164	1.41E-59	2.26E-57	124.84785
ARNTL2	2.580308	9.7129302	18.580543	2.49E-59	3.93E-57	124.28112
CFAP157	2.3736479	6.7714687	18.573459	2.69E-59	4.22E-57	124.20258
PMP2	-3.934361	1.3207851	-18.48966	6.85E-59	1.05E-56	123.27386
VEGFD	-4.069301	2.6681293	-18.46683	8.84E-59	1.34E-56	123.02098
FAM89A	2.1335225	8.5535048	18.465069	9.01E-59	1.36E-56	123.00146
ADAMTS12	3.7016124	9.0802554	18.41104	1.64E-58	2.42E-56	122.40327
ANLN	2.1906781	10.87324	18.39355	2.00E-58	2.92E-56	122.20969
GFRA2	-3.887048	4.469652	-18.37854	2.36E-58	3.39E-56	122.04361
SKA3	2.282446	9.6625338	18.3329	3.92E-58	5.56E-56	121.53874
SST	-6.041345	2.7963448	-18.31403	4.84E-58	6.81E-56	121.33006
BMP3	-6.591654	4.9692445	-18.2889	6.39E-58	8.93E-56	121.05222
LARGE2	4.4554402	9.7148616	18.25575	9.24E-58	1.28E-55	120.68592
HILPDA	2.4652932	9.9483942	18.251476	9.69E-58	1.33E-55	120.63869
SMPDL3A	-2.630983	9.4706966	-18.22677	1.27E-57	1.74E-55	120.36581
SH3TC2	3.6570066	8.1890519	18.207568	1.58E-57	2.14E-55	120.15369
OTX1	5.175403	5.9190311	18.185066	2.03E-57	2.73E-55	119.90523
MS4A10	-4.09465	1.1113811	-18.1476	3.07E-57	4.11E-55	119.49168
PI16	-5.857928	4.1180733	-18.10958	4.68E-57	6.18E-55	119.07225
TMEM100	-4.346136	4.514825	-18.09345	5.59E-57	7.30E-55	118.89437
TCEAL6	-2.439829	0.6475666	-18.07963	6.52E-57	8.45E-55	118.74192
CA2	-5.333375	10.354882	-18.03674	1.05E-56	1.35E-54	118.26919
CEPT2	2.2735972	8.4573993	18.028784	1.15E-56	1.46E-54	118.18146
MSX2	4.8013894	8.1597679	17.975755	2.06E-56	2.58E-54	117.59731
RXRG	-3.762641	2.2428402	-17.95553	2.58E-56	3.17E-54	117.37458
SLC51A	-3.43161	7.1681964	-17.94339	2.95E-56	3.60E-54	117.24101
TNXB	-4.201121	7.7077352	-17.93698	3.16E-56	3.85E-54	117.17047
CST1	7.1049055	7.8121962	17.931479	3.36E-56	4.06E-54	117.10987
TMEM132A	2.5723197	10.248687	17.91325	4.12E-56	4.94E-54	116.90927
SCARA5	-5.747506	6.4417227	-17.8719	6.50E-56	7.75E-54	116.45441
TP53INP2	-2.622112	10.562176	-17.85545	7.80E-56	9.25E-54	116.27349
NRXN1	-5.477355	2.776688	-17.85448	7.88E-56	9.29E-54	116.26277
PDE2A	-2.529716	6.9174892	-17.8129	1.25E-55	1.46E-53	115.8058
TCEAL5	-3.076527	1.1278776	-17.73815	2.85E-55	3.32E-53	114.98474
CD177	-7.142137	7.1117012	-17.69718	4.48E-55	5.18E-53	114.53506
LRRN2	-3.985643	6.5937916	-17.67298	5.85E-55	6.69E-53	114.26955
GLP2R	-4.338073	4.7640345	-17.66357	6.49E-55	7.38E-53	114.16641
VWA2	4.1528216	9.0097651	17.657553	6.93E-55	7.79E-53	114.10039
CLDN8	-7.60231	2.8416041	-17.59227	1.42E-54	1.58E-52	113.38476
STRA6	5.0072	7.37787	17.583451	1.57E-54	1.73E-52	113.28816
SLC6A6	2.7783412	11.730062	17.532752	2.74E-54	3.01E-52	112.73295
FAM227A	2.9281419	6.1626989	17.50167	3.86E-54	4.22E-52	112.39276
FEV	-4.445239	1.9124634	-17.49563	4.12E-54	4.48E-52	112.32671
PLCD1	-2.279462	9.3896676	-17.48609	4.58E-54	4.95E-52	112.22228
PRIMA1	-5.63217	3.9525734	-17.48333	4.72E-54	5.07E-52	112.19205
MAGEE2	-2.212662	0.7209204	-17.45099	6.74E-54	7.18E-52	111.83842
IQGAP3	2.0393325	11.129011	17.450733	6.75E-54	7.18E-52	111.83558
STRIP2	2.9656854	7.7581352	17.42765	8.70E-54	9.21E-52	111.58323
LIFR	-3.626987	6.5947273	-17.36663	1.70E-53	1.79E-51	110.91656
NAAA	-2.03211	10.175775	-17.35491	1.94E-53	2.01E-51	110.78849
CLDN23	-2.973793	9.1821824	-17.35346	1.97E-53	2.04E-51	110.77266
MCM10	2.136014	8.8184983	17.333126	2.46E-53	2.53E-51	110.55071
ENTPD5	-2.580963	11.002696	-17.32498	2.69E-53	2.74E-51	110.46184
DSCC1	2.247512	8.8615529	17.31928	2.86E-53	2.90E-51	110.3996
CYP27B1	2.3620877	7.0903605	17.289383	3.97E-53	4.01E-51	110.07339
ECRG4	-4.60092	2.9184965	-17.26817	5.01E-53	5.00E-51	109.84201
RSPO2	-4.579674	3.4673927	-17.25545	5.76E-53	5.72E-51	109.70335
CA1	-9.221221	6.055016	-17.25032	6.10E-53	6.02E-51	109.64744
KLK6	7.2693092	7.3796846	17.233436	7.33E-53	7.21E-51	109.46336
ADHFE1	-2.564528	4.3178557	-17.20238	1.03E-52	1.01E-50	109.12493
SMOX	2.406597	9.5267661	17.180442	1.31E-52	1.28E-50	108.88601
POLR1G	2.1188595	8.1919817	17.178624	1.34E-52	1.29E-50	108.86621
PDK4	-3.331672	9.2040693	-17.17058	1.46E-52	1.40E-50	108.77861
RNF152	-2.951608	7.8223886	-17.07921	3.97E-52	3.77E-50	107.78447
CTHRC1	3.80169	9.3974421	17.071744	4.30E-52	4.07E-50	107.70328
CFAP251	2.9669656	6.4242222	17.067476	4.51E-52	4.24E-50	107.65688
GSTM5	-2.929073	5.2432229	-17.06034	4.88E-52	4.54E-50	107.57933
DDIAS	2.3753362	8.2636635	17.040606	6.05E-52	5.58E-50	107.36484
GNG7	-2.848416	6.5944948	-17.00195	9.23E-52	8.43E-50	106.94494
MAPK15	4.1637142	7.0037209	16.998833	9.55E-52	8.69E-50	106.91109
COL10A1	6.7330514	7.9939483	16.997007	9.74E-52	8.82E-50	106.89126
RHPN1	3.3569402	9.4535361	16.996604	9.78E-52	8.82E-50	106.88688
WNT3	2.8549329	6.6905473	16.981993	1.15E-51	1.02E-49	106.72826
TRIM29	4.3432377	10.781226	16.947209	1.68E-51	1.48E-49	106.35076
AVPR1B	-2.307743	0.6859011	-16.89446	2.98E-51	2.59E-49	105.77872
KLHL35	3.7102589	7.1809265	16.874104	3.72E-51	3.19E-49	105.55808
ATG9B	3.5360993	7.0872823	16.865078	4.11E-51	3.51E-49	105.46029
CDC25B	2.0960385	12.32866	16.860634	4.31E-51	3.67E-49	105.41214
AHCYL2	-2.730799	11.532588	-16.79475	8.83E-51	7.44E-49	104.69878
SPHKAP	-2.210036	0.5807986	-16.74542	1.51E-50	1.26E-48	104.16513
TROAP	2.1136436	9.7066831	16.744983	1.52E-50	1.26E-48	104.16043
CADM2	-4.520322	2.0558902	-16.72412	1.90E-50	1.57E-48	103.93495
TMCC3	-2.381354	9.753196	-16.71702	2.06E-50	1.69E-48	103.85814
TEX11	-3.715998	3.7173076	-16.71628	2.07E-50	1.70E-48	103.85011
LEMD1	5.195105	5.8398854	16.703243	2.39E-50	1.95E-48	103.70929
PRKG2	-3.807804	5.0452116	-16.69788	2.53E-50	2.05E-48	103.65131
PLCE1	-2.089413	9.9618651	-16.69319	2.66E-50	2.15E-48	103.60068
OXTR	2.9660514	5.9778372	16.690663	2.74E-50	2.20E-48	103.57338
ANKRD13B	2.5988811	8.9807209	16.678718	3.12E-50	2.49E-48	103.44436
ELANE	-3.0616	1.5754682	-16.66139	3.76E-50	2.99E-48	103.25727
SFRP1	-5.604289	5.5496593	-16.6611	3.77E-50	2.99E-48	103.25409
CCN4	3.136198	7.7421734	16.622225	5.75E-50	4.48E-48	102.83455
CMA1	-4.348249	2.1337216	-16.5821	8.89E-50	6.87E-48	102.40184
ABCB11	-4.451477	2.4487795	-16.56943	1.02E-49	7.86E-48	102.26519
GRHL1	2.6758038	7.4876386	16.568378	1.03E-49	7.92E-48	102.25388
P2RX2	-3.274357	1.5280084	-16.54225	1.34E-49	1.02E-47	101.99389
XKR4	-4.183008	2.2634011	-16.5069	2.01E-49	1.52E-47	101.59163
RGS16	2.568797	9.1492603	16.497443	2.22E-49	1.67E-47	101.4898
TMEM220	-2.965517	6.6564318	-16.48888	2.44E-49	1.83E-47	101.39769
AQP8	-8.610908	6.2042464	-16.4654	3.15E-49	2.34E-47	101.14498
KCNJ14	2.3532525	6.3770134	16.447563	3.82E-49	2.82E-47	100.95312
PIRT	-4.554726	2.926311	-16.44319	4.00E-49	2.94E-47	100.9061
NEBL	2.3912069	11.559331	16.442315	4.04E-49	2.96E-47	100.89669
PTN	-2.775939	6.34432	-16.40252	6.21E-49	4.52E-47	100.46892
SCN9A	-4.094036	5.5341718	-16.40152	6.28E-49	4.55E-47	100.45819
JAM2	-2.632229	7.3454883	-16.39189	6.97E-49	5.03E-47	100.35467
ATP1A2	-5.316042	4.792656	-16.38971	7.13E-49	5.13E-47	100.33123
HTR1D	4.6668849	7.395717	16.373296	8.52E-49	6.10E-47	100.15497
CCDC69	-2.578636	9.3441245	-16.36047	9.78E-49	6.98E-47	100.01722
ENPP6	-3.322044	3.1681892	-16.35814	1.00E-48	7.11E-47	99.992262
SULT1A2	-3.977744	5.5652076	-16.35529	1.03E-48	7.30E-47	99.961631
GPAT3	-2.705375	8.0663658	-16.33314	1.31E-48	9.21E-47	99.723939
WDR62	2.1248299	9.0361066	16.319953	1.51E-48	1.06E-46	99.582399
EME1	2.1323421	7.2380469	16.279462	2.34E-48	1.62E-46	99.148154
NSG2	-4.54402	3.0037899	-16.27934	2.35E-48	1.62E-46	99.146822
METTL7A	-2.66281	10.607699	-16.26655	2.69E-48	1.85E-46	99.009737
MUSK	-3.713049	2.1859023	-16.22152	4.38E-48	2.96E-46	98.527399
SEMA6D	-3.382398	7.8469904	-16.22084	4.41E-48	2.97E-46	98.520139
GCG	-7.1011	3.4876795	-16.20044	5.49E-48	3.68E-46	98.301692
TPH1	-3.886154	4.5549402	-16.19722	5.68E-48	3.80E-46	98.267192
C2CD4A	4.4351603	8.8594036	16.169956	7.62E-48	5.07E-46	97.97551
SLC26A2	-4.627431	11.292322	-16.16335	8.18E-48	5.43E-46	97.904878
E2F7	2.3157149	8.0436033	16.157398	8.72E-48	5.77E-46	97.841191
NAP1L2	-3.823947	3.3399116	-16.1534	9.11E-48	6.00E-46	97.798486
LRRC3B	-2.112037	0.6890288	-16.1453	9.94E-48	6.50E-46	97.711865
NR3C2	-2.925352	9.4840764	-16.14011	1.05E-47	6.85E-46	97.65633
TMEM37	-3.232042	8.9836841	-16.12192	1.28E-47	8.28E-46	97.461946
GALR1	-2.936625	1.067938	-16.10742	1.49E-47	9.61E-46	97.307025
ABI3BP	-3.393059	7.5917345	-16.10468	1.54E-47	9.86E-46	97.277723
GUCA2A	-6.578177	7.98548	-16.08931	1.81E-47	1.16E-45	97.11352
KIF18B	2.0053993	9.5641194	16.083064	1.94E-47	1.23E-45	97.046846
SLC7A14	-4.00051	1.7793249	-16.07572	2.10E-47	1.33E-45	96.968421
FHL1	-3.147548	9.5541265	-16.06259	2.42E-47	1.52E-45	96.828269
CAVIN2	-2.888772	7.7759128	-16.06062	2.47E-47	1.54E-45	96.807269
[illegible]	[illegible]	[illegible]	[illegible]	[illegible]	[illegible]	[illegible]
